# Taxonomy, systematics and geographic distribution of ground frogs (Alsodidae, *Eupsophus*): a comprehensive synthesis of the last six decades of research

**DOI:** 10.3897/zookeys.863.35484

**Published:** 2019-07-11

**Authors:** Claudio Correa, Felipe Durán

**Affiliations:** 1 Departamento de Zoología, Facultad de Ciencias Naturales y Oceanográficas, Universidad de Concepción, Barrio Universitario S/N, P.O. Box 160-C, Concepción, Chile Universidad de Concepción Concepción Chile; 2 Magíster en Ciencias con mención en Zoología, Departamento de Zoología, Facultad de Ciencias Naturales y Oceanográficas, Universidad de Concepción, Barrio Universitario S/N, P.O. Box 160-C, Concepción, Chile Universidad de Concepción Concepción Chile

**Keywords:** Biogeography, diagnoses, ground frogs, literature review, southern South America, species groups

## Abstract

The genus *Eupsophus* (ground frogs) inhabits exclusively the temperate forests of southern South America (Chile and Argentina). The current delimitation of the genus was reached in the late 1970s, when only two species were recognized, but since then the number of described species steadily increased, reaching a maximum of 11 by 2012. Subsequent studies that applied explicit species delimitation approaches decreased the number of species to six in 2017 and raised it again to 11 the following year, including an undescribed putative species. Despite these taxonomic changes, the two species groups traditionally recognized, *roseus* and *vertebralis*, have been maintained. Another recent contribution to the taxonomy of the genus was the explicit recognition of the extremely high level of external phenotypic variation exhibited by species of the *roseus* group, which undermines the utility of some diagnostic characters. Here we provide a critical review of the extensive taxonomic and systematic literature on the genus over the last six decades, to examine the evidence behind the recurrent taxonomic changes and advances in its systematics. We also update and complete a 2017 review of geographic information, provide additional qualitative observations of external characters commonly used in the diagnoses of species of the *roseus* group, and reassess the phylogenetic position of a putative new species from Tolhuaca (Chile), which was not included in the last species delimitation study. The present review shows that: 1) there is no congruence between the patterns of phenotypic and genetic/phylogenetic differentiation among species of both groups; 2) in the *roseus* group, the intraspecific variation in some external characters is as high as the differences described among species; 3) there is little morphological and bioacoustic differentiation within species groups, and inconsistencies in the chromosomal evidence at the genus level; 4) under the latest taxonomic proposal (2018), species of the *roseus* group still lack consistent and reliable diagnoses and their distribution limits are poorly defined; and 5) the population from Tolhuaca represents an additional undescribed species under the most recent taxonomic framework. Finally, we discuss the implications of these findings for the taxonomy and biogeography of the genus, pointing out some areas that require further research to understand their patterns and processes of diversification.

## Introduction

**Figure 1.**
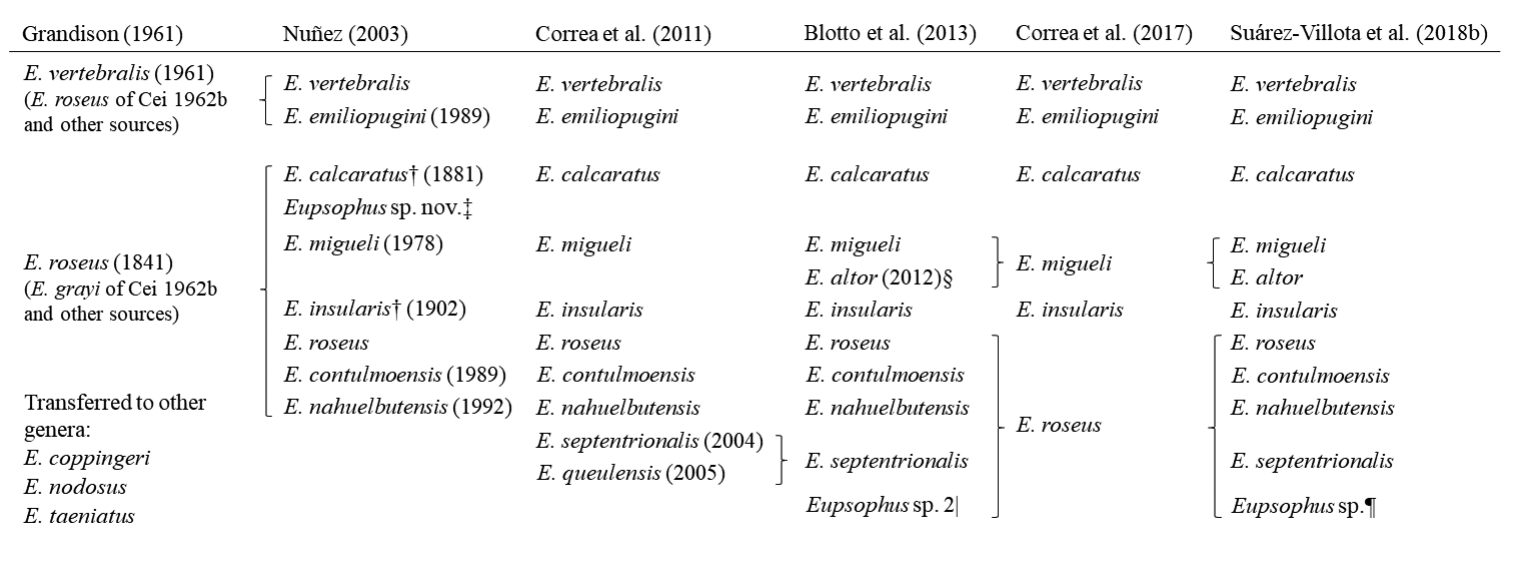
Composition of the genus *Eupsophus* between 1961 and 2018 according to several reviews and studies. Year of species description is provided in parentheses. Capurro (1958) and Cei (1958, 1960, 1962a, 1962b) recognized the same two species of Grandison (1961), but with different names (see comment in Cei 1962b). †Revalidated by Formas and Vera (1982) (removed from the synonymy of *E.roseus*). ‡Undescribed species from Isla Wellington (Chile), sister to *E.calcaratus*. §It appears as *Eupsophus* sp. 1 in Blotto et al. (2013). |Probable undescribed species from Tolhuaca (Chile), sister to *E.roseus*. ¶Putative species from Villarrica (Chile), sister to *E.roseus*.

Temperate forests of southern South America (Chile and Argentina) are home to a reduced but evolutionarily diverse group of amphibians (Formas 1979, Cei 1980, Correa et al. 2006, Blotto et al. 2013, Streicher et al. 2018). The most diversified anuran lineage of these forests is the family Alsodidae, which currently is represented there by two sister genera, *Alsodes* Bell, 1843 (19 species; Blotto et al. 2013, Frost 2019) and *Eupsophus* Fitzinger, 1843 (11 species; Suárez-Villota et al. 2018b). Only *Eupsophus* (members commonly referred to as “ground frogs”) is found exclusively in temperate forests, inhabiting mainly the forest floor (Rabanal and Nuñez 2008). Recently, a controversy about the number of species of *Eupsophus* has emerged in the literature (Correa et al. 2017, Suárez-Villota et al. 2018b), according to which there are six or eleven species, respectively. The 11 species of the last taxonomic proposal (Suárez-Villota et al. 2018b) are arranged into the two species groups traditionally recognized (Fig. 1): *roseus* (*E.roseus* (Duméril & Bibron, 1841), *E.calcaratus* (Günther, 1881), *E.insularis* (Philippi, 1902), *E.migueli* Formas, 1978, *E.contulmoensis* Ortiz, Ibarra-Vidal & Formas, 1989, *E.nahuelbutensis* Ortiz & Ibarra-Vidal, 1992, *E.septentrionalis* Ibarra-Vidal, Ortiz & Torres-Pérez, 2004, *E.altor* Nuñez, Rabanal & Formas, 2012, and a putative new species from Villarrica, Chile) and *vertebralis* (*E.vertebralis* Grandison, 1961 and *E.emiliopugini* Formas, 1989) (Formas 1991, Nuñez 2003, Blotto et al. 2013, Suárez-Villota et al. 2018b).

The genus *Eupsophus* has a long and complex taxonomic history. Among the currently valid species, the first two were described in the nineteenth century under other genera: *Cystignathusroseus* and *Cacotuscalcaratus* (see the dates of description in Fig. 1). Subsequently, several species were described under now disused generic names (*Borborocoetes*, *Borborocoetus*, *Cystignathus*; e.g., Philippi 1902), among which only Borborocoetus (Cystignathus) insularis is currently recognized. The use of the name *Eupsophus*, coined by Fitzinger (1843), only became widespread in the first half of the twentieth century, when it included species from other currently valid genera (*Alsodes*, *Batrachyla*, *Phrynopus*, *Thoropa*; e.g., Capurro 1958, Grandison 1961, Cei 1962a, 1962b, Lynch 1971). The current delimitation of the genus was achieved in the late seventies (Lynch 1978), when only two species were recognized (*E.roseus* and *E.vertebralis*). Since 1978, when *E.migueli* was described (Formas 1978a), the number of species progressively increased to 11 (*E.calcaratus* and *E.insularis*, both revalidated by Formas and Vera 1982, *E.contulmoensis*, *E.emiliopugini*, *E.nahuelbutensis*, *E.septentrionalis*, *E.queulensis* and *E.altor*), but one of them, *E.queulensis*, was synonymized with *E.septentrionalis* by Blotto et al. (2013). The number of species was reduced to six by Correa et al. (2017), but the more recent proposal (Suárez-Villota et al. 2018b) restored the previous taxonomy, adding an additional species not described; so currently the genus is composed of ten nominal species plus an undescribed one (see the taxonomic changes since 1961 in Fig. 1).

During the last six decades, morphometric, immunological, chromosomal, bioacoustic and molecular (allozymes, RFLPs and DNA sequences) approaches have been applied, separately or in combination, to the taxonomy and systematics of these frogs (reviewed by Nuñez 2003). Phylogenetic analyses with DNA sequences only have been performed since Nuñez (2003), but they have had a profound influence on the estimation of species diversity and evolutionary patterns of the genus, particularly of the *roseus* group. Two of these studies (Nuñez et al. 2011, Blotto et al. 2013) suggested that the species diversity of that group may be underestimated. Nuñez et al. (2011) indicated that *E.calcaratus* would represent a species complex composed of six groups of mitochondrial haplotypes “diagnostic of species lineages”, and at least one of them would represent a new species (Villarrica population, foothills of Chilean Andes, 39° 20'S). Blotto et al. (2013) tested the monophyly of the genus and its species groups and investigated the relationships among species, including all the species recognized at that time. They synonymized *E.queulensis*with *E.septentrionalis* and suggested that the population from Tolhuaca, also located in the Chilean Andean foothills (38°13'S), would correspond to an undescribed species related to *E.roseus*. More recently, Correa et al. (2017), applying several unilocus species delimitation analyses with mitochondrial sequences, proposed a new arrangement that reduced the species of the genus to six. Suárez-Villota et al. (2018b) rejected this arrangement using new samples, different molecular markers and several species delimitation analyses (unilocus and multilocus). They considered as valid the ten species recognized before 2017 and found support for recognizing the population of Villarrica as a putative species, although they did not include specimens from Tolhuaca. All these hypotheses, including the species status of Villarrica and Tolhuaca populations, have been supported exclusively by molecular phylogenetic evidence, without explicitly incorporating phenotypic characters.

The application of molecular approaches and integrative taxonomy to the discovery and delimitation of species has drastically changed our estimates of amphibian diversity at global and local levels (Catenazzi 2015). Recent systematic research on *Eupsophus* frogs illustrates this trend, as shown by the putative new species mentioned above (Nuñez et al. 2011, Blotto et al. 2013), the description of *E.altor*, where an integrative taxonomy approach was applied (Nuñez et al. 2012a), and the most recent taxonomic proposals (Correa et al. 2017, Suárez-Villota et al. 2018b), based on explicit species delimitation analyses. However, descriptions and diagnoses of *Eupsophus* have historically been based primarily on external and internal phenotypic characters (Nuñez 2003) and molecular data have been included in only two cases (*E.septentrionalis* and *E.altor*, both considered invalid by Correa et al. 2017). Correa et al. (2017) pointed out some weaknesses of the diagnoses of the species of the *roseus* group, recognizing also that there are no known phenotypic characters to support their own taxonomic proposal. Moreover, they reviewed the chromosome and bioacoustic evidence published for the genus, finding a scarce differentiation in the karyotypes and advertisement calls among species of the *roseus* group, which was one of the decisive arguments for choosing a conservative delimitation (i.e., fewer species) in this group. On the other hand, the taxonomic proposal by Suárez-Villota et al. (2018b) rests exclusively on species delimitation approaches with DNA sequences, assuming that such a proposal is completely consistent with the numerous previous taxonomic and systematic studies of the genus based on non-molecular evidence.

The last complete review of the taxonomy and systematics of the genus *Eupsophus* was Nuñez (2003), a doctoral dissertation that was not published in a peer-reviewed journal. That review presented a rather stable and uncontroversial view of the taxonomy of the genus, which at that time comprised eight species. Since that date, there have been several changes in the composition of the genus, specifically in the *roseus* group (summarized in Fig. 1). Correa et al. (2017) reviewed partially the taxonomy and geographic information of the genus, with a focus on the *roseus* group. These authors not only noted the weaknesses of the diagnoses of the species of that group, but also the problems that arise when comparing all the published chromosomal, bioacoustic and geographic information on the genus.

In this study, we synthesize the vast taxonomic and systematic literature of the genus to identify the evidence supporting the recurrent taxonomic changes. We extend the review of Correa et al. (2017) to the whole genus, adding other lines of evidence that have been applied to the *Eupsophus* taxonomy, and provide a more complete compilation of geographic information. We also add new qualitative observations of external characters of live adults of selected populations and reassess the phylogenetic position of a putative new species from Tolhuaca (Andean foothills of Chile; Blotto et al. 2013), which was not included in the last species delimitation study (Suárez-Villota et al. 2018b). We aim not only to provide a complete and updated summary of the taxonomic, systematic and geographic information of the genus, but also to highlight the incongruences among different lines of evidence that should be addressed by future taxonomic and systematic studies.

## Materials and methods

### Literature sources

#### Taxonomy and systematics

Our literature review was focused on (but not restricted to) taxonomic, genetic and phylogenetic studies in which phenotypic and/or genetic variation within and among *Eupsophus* species is described. As starting point, we considered the first reviews exclusively dedicated to the taxonomy of Chilean *Eupsophus*, Cei (1960), Grandison (1961) and Cei (1962a), because they combined several problematic taxa (e.g., the forms described by Philippi 1902) under that genus name. Although those reviews (and some previous ones, such as Capurro 1958 and Cei 1958) included some species currently considered members of other South American genera (*Alsodes*, *Batrachyla*, *Phrynopus*, *Thoropa*), information about the genus, in its current definition (e.g., Lynch 1978), is easily retrievable. The last complete review of the taxonomy and systematics of *Eupsophus* is the unpublished doctoral dissertation of Nuñez (2003), but recently Correa et al. (2017) partially reviewed the chromosome, bioacoustic and geographic information on the genus. Other taxonomic and/or systematic studies with wider taxonomic coverage (but that include several species of *Eupsophus*) are Díaz (1986), Correa et al. (2006), and Blotto et al. (2013). The latter also contains a synthesis of the recent systematics of *Eupsophus* and was the most comprehensive molecular phylogenetic study of the genus until Correa et al. (2017) and Suárez-Villota et al. (2018b). Descriptions and redescriptions of the ten nominal species recognized by Suárez-Villota et al. (2018b) are included in Duméril and Bibron (1841) (*E.roseus* as *Cystignathusroseus*), Günther (1881) (*E.calcaratus* as *Cacotuscalcaratus*), Philippi (1902) (*E.insularis* as Borborocoetus (Cystignathus) insularis), Grandison (1961) (*E.vertebralis* and *E.roseus*, the latter as *E.grayi*), Capurro (1963) (who proposed to recognize *E.insularis* as subspecies of *E.grayi*), Formas (1978a) (*E.migueli*), Formas and Vera (1982) (revalidation of *E.calcaratus* and *E.insularis*), Formas (1989) (*E.emiliopugini*), Ortiz et al. (1989) (*E.contulmoensis*), Ortiz and Ibarra-Vidal (1992) (*E.nahuelbutensis*), Nuñez (2003) (which includes somewhat different descriptions of the aforementioned eight species), Ibarra-Vidal et al. (2004) (*E.septentrionalis*), Veloso et al. (2005) (*E.queulensis*, synonymized with *E.septentrionalis* by Blotto et al. 2013), and Nuñez et al. (2012a) (*E.altor*). Other studies of *Eupsophus* with a taxonomic and/or systematic focus have used different approaches: Capurro (1963) (morphology), Formas (1978b) (karyotypes), Formas (1980) (karyotypes), Iturra and Veloso (1981) (karyotypes), Formas et al. (1983) (allozymes), Formas (1985) (calls), Fernández de la Reguera (1987) (morphometrics), Iturra and Veloso (1989) (karyotypes), Formas (1991) (karyotypes), Formas et al. (1991) (allozymes), Formas et al. (1992) (allozymes and morphometrics), Formas (1992) (karyotypes), Formas and Brieva (1992) (immunology), Formas (1993) (allozymes and morphometrics), Formas and Brieva (1994) (calls), Cuevas and Formas (1996) (karyotypes), Nuñez et al. (1999) (morphometrics and RFLPs), Cárdenas-Rojas et al. (2007) (larval morphology), Nuñez and Úbeda (2009) (larval morphology), Opazo et al. (2009) (calls), Lavilla et al. (2010) (morphology), Nuñez et al. (2011) (phylogeography using mitochondrial sequences), and Vera Candioti et al. (2011) (larval morphology).

#### Geographic distributions

We compiled literature records to define the geographic ranges of the 11 species recognized by Suárez-Villota et al. (2018b) and compared them with the most recent maps (Nuñez 2003, Rabanal and Nuñez 2008, Correa et al. 2017, and IUCN 2019). Locality data were obtained from the publications in which the species were described (see above) and from other sources (e.g., Webb and Greer 1969, Formas and Vera 1980, 1982, Formas et al. 1991, Nuñez et al. 1999, Úbeda 2000, Díaz-Páez and Nuñez 2002, Méndez et al. 2005, Ortiz and Ibarra-Vidal 2005, Asencio et al. 2009, Nuñez et al. 2011, Blotto et al. 2013, Núñez and Gálvez 2015, Correa et al. 2017, Suárez-Villota et al. 2018b). Distribution data and/or maps of older reviews (Cei 1960, 1962a, 1962b, Grandison 1961, Formas 1979) were carefully considered because the delimitations of the species at that time were quite different from the present. In addition, we reviewed all biological studies of the genus and other relevant sources about Chilean amphibians to collect additional geographic data.

#### Phenotypic observations

Correa et al. (2017) showed that the four characters most frequently included in the diagnoses of the species of the *roseus* group (body coloration pattern, iris color, lateral and dorsal snout profile, and shape of the end of the xiphisternum) vary at the intrapopulation level. Here, we provide additional examples of intrapopulation variation in the first three characters. The observations were made in two undescribed and two type localities (Valdivia, *E.roseus*, and Mehuín, *E.migueli*), including less than 20 live specimens per locality. All specimens were released at the same capture site after being photographed.

#### Phylogenetic analyses

Blotto et al. (2013) identified one specimen from Tolhuaca (foothills of Chilean Andes, ~38°S) as a probable undescribed species, sister to *E.roseus*. Correa et al. (2017) included the same specimen and other samples from near Villarrica (as representatives of the area where there would be another undescribed species according to Nuñez et al. 2011) in their phylogenetic and species delimitation analyses, finding support for the inclusion of all of them into a redefined *E.roseus*. Suárez-Villota et al. (2018b) included specimens from Villarrica, but not from Tolhuaca in their species delimitation analyses, so the reciprocal relationships between both populations and the taxonomic status of the latter currently are not clear. Here we address both issues, using the two coding mitochondrial fragments included in common by Blotto et al. (2013), Suárez-Villota et al. (2018a, b): cytochrome b (cytb) and cytochrome c oxidase subunit I (COI). We concatenated the sequences of both fragments, totaling 147 specimens representing the ten currently recognized species and the two undescribed taxa (Villarrica and Tolhuaca). The sequences of both genes differ in length between studies, so an initial alignment was obtained with blocks of gaps at the ends of the genes. We obtained an alternative alignment by cutting those extremes. Two schemes to apply nucleotide evolution models were used in both alignments: considering each gene fragment as a partition or each position of the codons as a distinct partition within each fragment (six partitions). Sequences were aligned with Muscle v3.5 (Edgar 2004) and then inspected by eye. Phylogenetic relationships were estimated through a Bayesian inference (BI) method with a Markov Chain Monte Carlo algorithm, performed with the program MrBayes v3.2.6 (Ronquist et al. 2012). A General Time Reversible, plus gamma and proportion of invariable sites model was independently applied to each fragment/partition, using also a reversible jump method. Two independent BI analyses (each consisting of two groups of four chains that ran independently) applying that method were run for 10 million generations, sampling every 1000^th^ generation. The first 25% of generations were conservatively discarded as burn-in after observing the stationarity of ln-likelihoods of trees in Tracer v1.7 (Rambaut et al. 2018). Convergence and mixing of chains were assessed examining values of average standard deviation of split frequencies, and expected sampling sizes and potential scale reduction factors for all parameters. One specimen of *Alsodesnorae* of Suárez-Villota et al. (2018b) was used as outgroup (MK180951, cytb; MK181499, COI).

## Results

### Literature review

#### Changes in the content of the genus and species groups

The reviews by Cei (1960, 1962a) and Grandison (1961) are fundamental for the recent taxonomy of *Eupsophus*, because they combined several invalid (for example, several forms of *Cystignathus* and *Borborocoetus* of Philippi 1902) and now valid species (*E.calcaratus* and *E.insularis*) into two taxa, which represent the two species groups currently recognized (Fig. 1; see below). However, since the description of *E.migueli* (Formas 1978a), the number of species increased from three to eleven (with *E.altor*), most of them derived from or closely related to *E.roseus*. One additional species from Isla Wellington (southern Chile), closely related to *E.calcaratus*, was proposed by Nuñez (2003), though it was never named or formally described (Fig. 1; Blotto et al. 2013 included specimens from Isla Wellington, showing that they belong to *E.calcaratus*). All descriptions and revalidations (in the case of *E.calcaratus* and *E.insularis*) were primarily motivated by observations of differences in external morphological characters and in some cases also internal ones. Other types of characters were added in some descriptions and diagnoses (see Table 1), but only exceptionally additional evidence was obtained subsequently to reinforce the distinction of some species (e.g., the karyotype of *E.migueli*, Iturra and Veloso 1981). Another important change was the synonymization of *E.queulensis* with *E.septentrionalis* (Blotto et al. 2013), which resulted in ten formally recognized species until 2017. That year, Correa et al. (2017) proposed to synonymize *E.contulmoensis*, *E.nahuelbutensis* and *E.septentrionalis* with *E.roseus*, and *E.altor* with *E.migueli*, thus reducing from ten to six the species of the genus (Fig. 1). These authors suggested that part of the diversity of species previously recognized was due to the excessive importance attributed to non-fixed morphological differences in certain populations. These last synonymizations were reverted by Suárez-Villota et al. (2018b), who revalidated the same ten species recognized by 2017 plus one not described from Villarrica, Chile (Fig. 1), although they did not include specimens from Tolhuaca, Chile (*Eupsophus* sp. 2 of Blotto et al. 2013, Fig. 1). The division of *Eupsophus* into two species groups, *roseus* and *vertebralis* (Fig. 1), already implicit in the reviews of Cei (1960, 1962a) and Grandison (1961), it was first formally proposed by Formas (1991) based on karyotype differences. This division has been supported by cumulative morphological (Fernández de la Reguera 1987, Nuñez 2003), chromosomal (Formas 1980, Formas 1991), bioacoustic (advertisement calls; Formas 1985, Formas and Brieva 1994), genetic (allozymes; Formas et al. 1983) and immunological evidence (Formas and Brieva 1992). More recently, molecular phylogenetic analyses with DNA sequences have ratified the reciprocal monophyly and high genetic divergence between those groups (Nuñez 2003, Correa et al. 2006, Blotto et al. 2013, Correa et al. 2017, Suárez-Villota et al. 2018a, b).

#### Diagnostic characters

Correa et al. (2017) summarized the diagnostic characters of nine species of the *roseus* group (the eight species currently recognized plus *E.queulensis*). They extracted the information mainly from the original diagnoses, but also used other two sources for *E.roseus*, *E.calcaratus* and *E.insularis*, since the original descriptions and diagnoses of these species are very brief and were made under generic names no longer used. The two additional sources are Formas and Vera (1982), where *E.calcaratus* and *E.insularis*are revalidated, and Nuñez (2003), which contains partially different diagnoses for the eight species recognized at that date. The summary of Correa et al. (2017) highlighted several general deficiencies of the diagnoses of the species of the *roseus* group: 1) in some cases, characters that varied in the type series were used; 2) the great heterogeneity in number and type of characters used, which makes it difficult to identify the differences among the species; and 3) the four characters most frequently included in the diagnoses vary widely at the intraspecific level. Here (Table 1), we expand the summary table of Correa et al. (2017) to include the species of the *vertebralis* group and reorder the species according to the taxonomy and phylogenetic hypothesis of Suárez-Villota et al. (2018b). Table 1 allows to compare the diagnostic differences between species within groups, showing that the diagnoses are heterogeneous in the number of characters and level of detail, so they are scarcely comparable, regardless of the taxonomic scheme used (Correa et al. 2017 or Suárez-Villota et al. 2018b). In particular, diagnoses of sister species do not contain characters in common (*E.migueli* and *E.altor*) or these could be differentiated only by the body coloration pattern (*E.contulmoensis* and *E.nahuelbutensis*, *E.vertebralis* and *E.emiliopugini*), which has been described as variable in most species (see Correa et al. 2017 and the section Phenotypic observations).

**Table 1.** Phenotypic, karyotypic and genetic characters included in the diagnoses of the ten nominal species of *Eupsophus* currently recognized (Suárez-Villota et al. 2018b; Fig. 1). This is a modified version of the table of Correa et al. (2017) with two main changes: the two species of the *vertebralis* group were added, and the diagnoses of *E.queulensis* and *E.septentrionalis* were combined. Species are ordered by species group and then by their phylogenetic affinities according to the hypothesis of Suárez-Villota et al. (2018b). Empty cells indicate that character was not included in the respective diagnosis. The V in parentheses indicates that the character was described as variable in the type series. Character states are described such as they appear in the original sources. Diagnoses of *E.roseus*, *E.calcaratus* and *E.insularis* include characters of their original descriptions and/or later redescriptions (see the text).

character	* E. calcaratus *	* E. roseus *	* E. contulmoensis *	* E. nahuelbutensis *	*E.septentrionalis*† (including *E.queulensis*)	* E. insularis *	* E. migueli *	* E. altor *	*E.vertebralis*‡	*E.emiliopugini*§
**Body coloration**		body with brown tones on a pink background; transparent or whitish ventral area	dark purple dorsal pigmentation and bright yellow belly (V)	white belly with dark brown marmorino (V)	dark brown to blackish on a light gray to leaden background (V); *E.queulensis*: two melanic dots on dorsal region and reddish ventral surfaces (V)	dark brown with irregular yellow spots on the dorsum and legs (V)	dark venter and white brilliant irregular spots (V)		usually with a vertebral band which may be as broad as the distance between the nares (V)	a distinctive olive-green band between the eyes
**Color of upper part of iris**	bronze-yellow	orange	bronze-yellow		light yellow; *E.queulensis*: yellow		bronze-yellow			
**Shape of snout**	pointed in dorsal and lateral view, noticeably protruding over the lower jaw|			sloping in lateral view (V)	*E.queulensis*: truncate in lateral view				broadly rounded	
**Xiphisternum**				ample in its middle portion and rounded at its extreme	*E.queulensis*: without a notch	truncated and slightly notched	notched			
**Canthus rostralis**		thin and short		ample and extended						
**Cloacal fold**				marked						
**Relative position of epicoracoids**		right over left			*E.queulensis*: left epicoracoid superimposed to the right one					
**Carpal tubercles**			inner palmar tubercle prominent		*E.queulensis*: prominent external and internal tubercles				subarticular tubercles prominent	
**Tips of toes**	rounded and prominent									
**Other osteological characters**¶	prevomers in narrow contact	vomerine teeth arranged in a transverse row			skull morphology#	prevomerine teeth below the choanae			vomerine teeth in two, only slightly curved groups	
**Karyotype**		eight pairs of biarmed chromosomes			*E.queulensis*: heteromorphic sexual chromosomes, and secondary constriction at the fourth pair		16 acrocentric chromosomes			
**Allozymes**					allozyme pattern# (V)					
**Reproductive traits**								early winter breeding season and terrestrial tadpoles		
**Advertisement call**								spectral elements reaching 20 kHz		mating call with two notes
**Genetic divergence**								nine nucleotide site substitutions in the mitochondrial control region from *E.migueli*#		

†We add the diagnosis of *E.queulensis* because it includes a greater number of characters. ‡The original diagnosis of *E.vertebralis* (Grandison 1961) is very extensive, but based exclusively on external morphology (e.g., characteristics of the skin, ears, and limbs), so here we only included those characters comparable with other species; Nuñez (2003) indicated that the diagnosis of Grandison (1961) also included specimens of *Alsodes*, but he did not provide further details to support this assertion. §Formas (1989) included the adult size in its diagnosis to differentiate it from its sister species *E.vertebralis*; however, there is a high degree of superposition in male and female sizes between both species (Table 1 of Formas 1989). |Formas and Vera (1982) used this character to differentiate *E.calcaratus* from *E.roseus*, but they did not describe the snout profile of *E.roseus* (they only showed a drawing of the head in lateral profile). ¶To simplify the table, we reunite in this miscellaneous category a series of osteological details of the skull that have been included occasionally in the diagnoses. #These are not character states, but we transcribed them as they appear in the original diagnosis.

#### Variation in diagnostic characters

Correa et al. (2017) showed, using literature information and observations of live specimens, that the four characters most frequently included in diagnoses (body coloration, color of upper part of iris, shape of snout and shape of the end of the xiphisternum) vary within species. Here we summarize the information used by those authors and add some additional details from the literature. The first comprehensive reviews of the genus (Cei 1960, 1962a, Grandison 1961) already mentioned, although briefly, that body coloration patterns vary at intrapopulation level in species of the *roseus* group. However, these type of observations did not prevent the coloration pattern (dorsal and/or ventral) from being later included as a diagnostic character for several species of the group (Table 1). Moreover, according to their descriptions, body coloration varies in *E.calcaratus* (Formas and Vera 1982), *E.emiliopugini* (Formas 1989) and *E.altor* (Nuñez et al. 2012a; see their fig. 5). Another characteristic that contributes to the variation of the dorsal coloration patterns is a mid-dorsal (vertebral) line of whitish or yellowish color, which may be present or absent, and vary in length and width. This vertebral line is more frequent in the two species of the *vertebralis* group (Cei 1962b, Grandison 1961, Formas 1989), but also has been reported in some specimens of *E.migueli* (Formas 1978a), *E.calcaratus* (Formas and Vera 1982), *E.contulmoensis* (Ortiz et al. 1989), *E.nahuelbutensis* (Ortiz and Ibarra-Vidal 1992) and *E.septentrionalis* (Ibarra-Vidal et al. 2004, Veloso et al. 2005; see also Fig. 4B). Correa et al. (2017) discussed the possible causes and practical consequences of the variation of the body coloration patterns, adding several examples with live specimens of the *roseus* group (see their Supporting Information). There are also previous literature records of variation in the other three characters mentioned. The coloration of the iris has been included recurrently in the descriptions and diagnoses of the species of the *roseus* group, so it was considered a useful character to distinguish certain species (Table 1). In contrast, the iris of both species of the *vertebralis* group is very similar, uniformly reticulated in black and yellowish (Nuñez 2003). Iris coloration appears to be a less variable trait, because there are only a couple of references of intraspecific variation in the literature. Nuñez (2003) suggested indirectly that there is variation in this trait: the iris color of *E.calcaratus* and *E.nahuelbutensis* is “generally” yellow, whereas that of *E.roseus*, *E.migueli*, and *E.contulmoensis* “can be” orange. Moreover, Nuñez et al. (1999) mentioned that the typical copper-colored upper part of the iris of *E.roseus* is also observed occasionally in specimens of *E.calcaratus*, which otherwise is bronze-yellow. The snout profile also has been included in several diagnoses of species of both groups (Table 1). For example, the snout profile, both in dorsal and lateral view, was one of the few characters used by Formas and Vera (1982) to differentiate *E.calcaratus* from *E.roseus*. Only in the case of *E.nahuelbutensis* this character was described as variable in the type series (some paratypes had the snout rounded, Ortiz and Ibarra-Vidal 1992). Another instance of intraspecific variation stems from the synonymy of *E.queulensis* with *E.septentrionalis*, since the shape of the snout was described as truncate in the former (Veloso et al. 2005) and short and rounded in lateral profile in the latter (Ibarra-Vidal et al. 2004; Table 1). Correa et al. (2017) gave examples of intrapopulation variation of iris coloration and snout profile in live specimens of several populations, including individuals of the type localities of *E.roseus* and *E.altor*, showing that these characters are not useful to diagnose the species of the *roseus* group. We provide additional examples of variation of body and iris coloration and snout profile with specimens of four localities, including the type localities of *E.roseus* and *E.migueli* (section Phenotypic observations). The shape of the distal end of the xiphisternum is the osteological character most frequently included in descriptions and diagnoses (Table 1), where it has been implicitly considered as fixed. According to the literature, the xiphisternum of most species is rounded and unnotched (*E.roseus*, *E.calcaratus*, *E.vertebralis*, *E.contulmoensis*, *E.nahuelbutensis*, *E.septentrionalis*, *E.queulensis*, and *E.altor*), but in *E.insularis* it is truncated and slightly notched (Capurro 1963, Formas and Vera 1982; although in this last study it was drawn as unnotched), and in *E.migueli* it is notched (Formas 1978a) (this character has not been described in *E.emiliopugini*). However, one study (Díaz 1986) examined the form of the xiphisternum in a significant number of specimens from the type localities of *E.roseus* (Valdivia, *N* = 37) and of *E.migueli* (Mehuín, *N* = 45), finding four types of xiphisternum (rounded, pointed, notched and seminotched) in *E.migueli* and three in *E.roseus* (notched condition was not found). Although in both species the rounded xiphisternum was the most frequent condition, this example demonstrates that intrapopulational variation in osteological characters may be detected when a large number of specimens is examined. Nuñez (2003) mentioned that some osteological characters vary at intra- and interspecific levels (for example, the relative position of epicoracoids, which has been included in the diagnoses of two species, Table 1), though which species display the variation were not specified by the author.

#### Morphometric studies

Morphometric approaches have usually been used to infer, implicitly or explicitly, the relationships among species or to discriminate (or validate) them. Also, they have been used in conjunction with allozymes (see below) to evaluate explicitly the agreement between morphological and genetic evolution in the genus (Formas et al. 1983, Formas et al. 1992, Formas 1993). The first comprehensive reviews (Grandison 1961, Cei 1962a) contain measurements and/or indices (ratios) of body, head and hind legs of adults of only two species of *Eupsophus* (equivalent to the two species groups) and the other species (*Alsodes* spp., *Batrachylataeniata*) that the genus contained at that time. Cei (1962a) described morphometric differences between continental and insular (Chiloé Island) populations of *E.grayi* (equivalent to the current *roseus* group), but in those groups of populations he mixed several species that were described later. Subsequent studies on adults have applied multivariate statistical techniques (mainly principal components and discriminant analyses), but they have been carried out with a small number of species (no more than four species per study; *E.nahuelbutensis* and *E.septentrionalis* have not been included in any study) and populations (no study included more than one population per species). Despite these limitations, morphometric differences have been observed between the species groups (Fernández de la Reguera 1987), and not within them (Formas et al. 1983, Díaz 1986, Fernández de la Reguera 1987, Formas et al. 1992, Formas 1993, Nuñez et al. 1999, Nuñez et al. 2012a). In particular, some species of the *roseus* group are morphometrically indistinguishable from each other (*E.roseus*, *E.migueli*, and *E.altor*; Díaz 1986, Nuñez et al. 2012a). Similarly, the only comparative morphometric study of tadpoles, Nuñez and Úbeda (2009), showed a clear differentiation between species groups (*E.vertebralis* and *E.emiliopugini* versus *E.roseus* and *E.nahuelbutensis*), but scarce differences within them.

#### Chromosomal studies

The karyotypes of nine of the ten species of *Eupsophus* currently recognized are shown in Table 2, ordered by species group and date of description (that of *E.nahuelbutensis* has not been described, although Nuñez 2003 pointed out that it has 30 chromosomes). Species groups are characterized by different numbers of chromosomes (30 in the *roseus* group, 28 in the *vertebralis* group; Nuñez 2003, Veloso et al. 2005) and three species present heteromorphic sex chromosomes (*E.migueli*, Iturra and Veloso 1981; *E.insularis*, Cuevas and Formas 1996; and *E.septentrionalis*, Veloso et al. 2005). In *E.roseus* the sex chromosomes do not differ in form, but can be distinguished by their constitutive heterochromatin patterns (Iturra and Veloso 1989). Correa et al. (2017) noted that different authors described different karyotypes for the same population in several species, without reporting variation among the specimens used, even though in most studies more than one was included (in some cases more than ten, e.g., Formas 1978a, 1978b, Cuevas and Formas 1996). Correa et al. (2017) argued that these differences are due to observer biases, which is consistent with the information of the karyotypes summarized in Table 2, where karyotypes of the same species obtained by several authors, from the same (e.g., *E.roseus*, *E.migueli*) or several localities (e.g., *E.roseus*, *E.calcaratus*) can be compared. Almost all karyotypes of the same species described by different authors differ in chromosomal morphology and position of the secondary constriction, and even in the presence or absence of this last structure (*E.vertebralis* and *E.emiliopugini*), so that intrapopulation and/or intraspecific variation is revealed only when different studies are compared. The levels of variation in chromosome morphology and position of the secondary constriction within a same species (considering all studies by different authors) are as high as the levels of variation observed among species of the same group (e.g., between *E.migueli* and *E.insularis*, or between *E.roseus* and *E.contulmoensis*; Table 2). The discovery of heteromorphic sex chromosomes in *E.migueli* (Iturra and Veloso 1981) is another example of inconsistent descriptions of karyotypes of the same population and species, since they were not observed in previous studies of the species (Bogart 1970, Formas 1978a, 1978b; Table 2). Differences in chromosome morphology are not due to methodological issues, since all studies followed Levan et al. (1964) to determine the position of the centromere and Bogart (1970, 1973) to determine the relative lengths of the chromosomes, so we agree with the suggestion of Correa et al. (2017) that many of the differences among studies are observer-dependent.

**Table 2.** Summary of karyotypes described in *Eupsophus*. Species are ordered by group (*roseus* and *vertebralis*) and then by year of description and locality, considering the current taxonomy (Suárez-Villota et al. 2018b; Fig. 1). Number of samples (f: females, m: males, j: juveniles) for obtaining the karyotypes are indicated (when specified), although in some studies is not clear how many specimens were used (indicated with a question mark). Reported morphology of each chromosome (pairs 1–15; m: metacentric; sm: submetacentric; st: subtelocentric; t: telocentric), diploid number (2n) and fundamental number (*FN*) are also indicated. An asterisk indicates the chromosome bearing the secondary constriction. In several cases, chromosomal morphology was not described in the text or was described with ambiguity, so this information was inferred from the original figures (indicated with a question mark). Heteromorphic chromosomes (pair 14) have been described for three species and imply different chromosome morphology and fundamental number between sexes (both telocentric in females, *FN* = 44; metacentric and telocentric in males, *FN* = 45). Veloso et al. (2005) summarized the information of the karyotypes of the genus without specifying the source or the number of samples.

species	source	locality	sample size	1	2	3	4	5	6	7	8	9	10	11	12	13	14	15	2n	*FN*
* roseus *	Formas (1978b)	Valdivia (city)	12m, 6f	m	sm	st*	t	t	m	m	t?	sm?	t	t?	m?	t	m	t	30	46
* roseus *	Formas (1978a)	near Valdivia (city)	2m, 14f	m	st*	st	t	t	m	m	m	t	m	t	t	m	t	t	30	46
* roseus *	Iturra and Veloso (1989)	Valdivia (city)	4m, 4f?	m	sm*	st	t	t	t	m	m	m	t	t	m	t	m	t	30	46
* roseus *	Formas (1978b)	Fundo San Martín	12m, 11f	m	st	st	t	t*	t	m	m	m	t	t	t	m	t	m	30	46
* roseus *	Veloso et al. (2005)	not specified	–	m	sm*	st	t	t	t	m	m	m	t	t	m	t	m	t	30	46
*calcaratus*†	Barrio and Rinaldi de Chieri (1971)	Puerto Blest (Río Negro, Argentina)	3m	–	–	–	–	–	–	–	–	–	–	–	–	–	–	–	30	–
*calcaratus*†	Veloso et al. (1974)	P.N. Vicente Pérez Rosales	5m, 2f, 1j	m	st*	st	t	t	t	m	m	m	t	t	m	t	m	t	30	46
* calcaratus *	Formas (1980)	La Picada, Cordillera Pelada and P.N. Puyehue	3m, 7f; 11m, 1f; 2m	m	sm*	st	t	m	t	m	m	t	m	t	t	m	t	t	30	46
* calcaratus *	Veloso et al. (2005)	not specified	–	m	sm	st*	t	m	t	m	m	t	m	t	t	m	t	t	30	46
* insularis *	Cuevas and Formas (1996)	Isla Mocha	11m, 9f	m	sm*	st	m	t	m	t	t	m	t	m	t	t	m/t	t	30	45/44
* insularis *	Veloso et al. (2005)	not specified	–	m	sm	st*	m	t	m	t	t	m	t	m	t	t	m/t	t	30	45/44
*migueli*†	Bogart (1970)	Mehuín	2m, 1f	m	st*	st	t	t	t	m	m	m	t	t	m	t	t	t	30	44
*migueli*†	Formas (1978b)	Mehuín	23m, 4f	m	sm*	st	t?	t?	m	m	m	t?	m	t	t	t	t	t	30	44
* migueli *	Formas (1978a)	Mehuín	7m, 3f	m	st*	st	t	m	m	t	m	t?	t	t	t	t	t	t	30	44
* migueli *	Iturra and Veloso (1981)	Mehuín	14m, 10f	–	–	–	–	–	–	–	–	–	–	–	–	–	–	–	30	45/44
* migueli *	Iturra and Veloso (1989)	Mehuín	4m, 4f?	m	sm*	st	t	t	t	m	m	m	t	t	m	t	m/t	t	30	45/44
* migueli *	Veloso et al. (2005)	not specified	–	m	sm*	st	t	t	t	m	m	m	t	t	m	t	m/t	t	30	45/44
* contulmoensis *	Formas (1992)	M.N. Contulmo	5m, 3f?	m	st	sm*	t	t	m	m	t	t	m	m	t	t	m	t	30	46
* contulmoensis *	Veloso et al. (2005)	not specified	–	m	st	st*	t	t	m	m	t	t	m	m	t	t	m	t	30	46
*septentrionalis*‡	Veloso et al. (2005)	R.N. Los Queules	1m, 1f	m	st	st	t*	t	m	t	m	sm	t	t	m	t	m/t	t	30	45/44
* altor *	Nuñez et al. (2012a)	not specified	1h	m	sm*	st	t	m	t	t	t	m	m	m	t	t	t	t	30	44
* vertebralis *	Bogart (1970)	Mehuín	2m	sm	m	t	st	st	st	sm	m	m	m	sm	sm	m	m		28	–
* vertebralis *	Formas (1991)	Mehuín	9m, 1f	m	st	m	st	st*	st	sm	m	m	m	m	m	t	m		28	54
* vertebralis *	Veloso et al. (2005)	not specified	–	m	st	m	st	st	st	sm	m	m	m	m	m	t	m		28	56§
*emiliopugini*|	Veloso et al. (1974)	P.N. Vicente Pérez Rosales	1m, 1f	m	m	st	st	st	t	sm	sm	sm	m	m	sm	m	m		28	54
* emiliopugini *	Formas (1991)	Puntra	4m, 2f	m	st	m	st	st*	st	sm	m	m	m	m	m	m	m		28	56
* emiliopugini *	Veloso et al. (2005)	not specified	–	m	st	m	st	st	st	sm	m	m	m	m	m	m	m		28	56

†As *E.roseus*. ‡As *E.queulensis*. §According to the chromosomal morphology, it should be 54, but here the original figure of the Table 2 from Veloso et al. (2005) is reported. |As *E.vertebralis*.

Bioacoustic studies

Vocalizations of nine nominal species of both species groups have been described (Table 3; summarized by Nuñez 2003 and Correa et al. 2017). The vocalizations emitted more frequently by males are advertisement calls (called type A or short calls; Formas 1985, Formas and Brieva 1994, Penna and Veloso 1990), which have been described for most species. The difference in the temporal and spectral (frequencies) structure of the advertisement calls is one of the lines of evidence that has been used to support the division of the genus into two groups (Formas 1985, Formas and Brieva 1994, Nuñez 2003). Also, long calls (> 2.7 seconds; type B of Formas 1985) are emitted by males of some species of the *roseus* group, which could correspond to territorial or encounter calls (Formas 1985, Penna and Veloso 1990), but these calls have been described only in *E.migueli* (Formas 1985, Penna and Veloso 1990) and *E.roseus* (Penna and Veloso 1990) (Table 3). Another type of call described in the *roseus* group is an aggressive call recorded occasionally in *E.calcaratus* and *E.roseus* (Márquez et al. 2005). Short advertisement calls are structurally very similar among species of the *roseus* group: all calls consist of only one note and ranges of temporal and spectral parameters overlap extensively among species (Table 3; see comments in Formas and Brieva 1994 and Correa et al. 2017). Formas and Brieva (1994) noted only differences in the intervals among harmonics among species of the *roseus* group: *E.contulmoensis* and *E.insularis* have harmonics at about 500 Hz, while *E.calcaratus*, *E.migueli* and *E.roseus* show harmonics at about 1000 Hz intervals. Instead, the advertisement calls of both species of the *vertebralis* group differ in notes per call, although the other parameters show a high degree of overlap (Formas 1989, Nuñez 2003). Table 3 contains the parameters most commonly used in the descriptions of *Eupsophus* vocalizations, but other parameters have been reported in some species: for example, pulses per second in *E.roseus* and *E.vertebralis* (as *E.vittatus*, Formas and Vera 1980), and notes per second and note duration in *E.vertebralis* and for long calls of *E.roseus* and *E.migueli* (Penna and Veloso 1990). More recently, the maximum frequency was included in the diagnosis of *E.altor* (Nuñez et al. 2012a) to differentiate it from *E.roseus* and *E.migueli*: this parameter surpasses 20 kHz in *E.altor*, while in the other two species it does not exceed 15 kHz. Correa et al. (2017) argued that this parameter would be the only diagnostic difference to distinguish *E.altor* from *E.migueli*, but they considered it insufficient to support the validity of *E.altor*. Variation in frequency modulation patterns of short advertisement calls have been described in *E.calcaratus* (Márquez et al. 2005), *E.roseus* (Márquez et al. 2005) and *E.septentrionalis* (as *E.queulensis*; Opazo et al. 2009).

**Table 3.** Parameters most commonly used to describe the vocalizations of nine of the ten species of *Eupsophus* currently recognized (see Fig. 1; vocalizations of *E.nahuelbutensis* has been not described). Species are ordered by group (*roseus* and *vertebralis*) and then by year of description. Mean and/or range (in parentheses after the means) of each parameter (N/C: notes per call; RR: repetition rate; CL: call length; PPN: pulses per note; FF: fundamental frequency; DF: dominant frequency) are given.

species	source	locality	N/C	RR (calls/min)	CL (ms)	PPN	FF (Hz)	DF (Hz)
* E. roseus *	Formas and Vera (1980)	Huachocopihue	1	64 (60-72)	200 (190-210)	17 (15-20)	-	2200 (1600-2900)
* E. roseus *	Penna and Veloso (1990)	Valdivia	1	25.1 (11.1-60)	105 (70-160)	-	-	1291 (1250-1350)
*E.roseus* (long call)	Penna and Veloso (1990)	Valdivia	32.1 (8-47)	10.2 (9.3-11.2)	2730 (650-4000)	-	-	1390 (1220-1470)
* E. roseus *	Márquez et al. (2005)	Lago Tinquilco	1	-	158 (124-235)	-	633 (346-1019)	1871 (1503-2167)†
* E. calcaratus *	Formas (1985)	Puntra	1	19 (16-25)	190 (150-210)	-	-	1100-2700‡
* E. calcaratus *	Márquez et al. (2005)	La Picada	1	-	192 (112-262)	-	776 (447-1104)	2157 (1805-2407)†
* E. insularis *	Formas and Brieva (1994)	Isla Mocha	1	7.8 (4-12)	160 (140-180)	-	-	1500-2100
* E. migueli *	Formas (1985)	Mehuín	1	6 (3-8)	240 (200-350)	-	450 (390-987)§	1835§ (1500-2500)
*E.migueli* (long call)	Formas (1985)	Mehuín	24 (19-33)	6 (5-8)	3400 (2700-4400)|	4-7¶	-	900-1500
* E. migueli *	Penna and Veloso (1990)	Mehuín	1	4.2 (2.4-6.6)	208 (160-260)	-	-	1633 (1170-1820)
*E.migueli* (long call)	Penna and Veloso (1990)	Mehuín	12.3 (4-23)	5.4 (2.4-8.4)	1072 (300-2160)	-	-	1532 (1210-2000)
* E. contulmoensis *	Formas and Brieva (1994)	M.N. Contulmo	1	23.3 (15-34)	180 (150-200)	-	-	1100-2000
*E.septentrionalis*#	Opazo et al. (2009)	R.N. Los Queules	1	-	135 (46-182)	-	-	1818 (1464-2326)†
* E. altor *	Nuñez et al. (2012a)	Parque Oncol	1	-	336 (290-360)††	-	756 (304-1298)††	1882 (1317-2098)
*E.vertebralis*‡‡	Formas and Vera (1980)	Mehuín	5 (4-6)	4 (2-10)	600 (400-800)	15 (11-23)	-	1900 (1100-2500)
* E. vertebralis *	Formas (1989)	Mehuín	5 (4-6)	-	89 (62-187)	15.9 (11-23)	-	1154 (600-1680)
* E. vertebralis *	Penna and Veloso (1990)	Mehuín	5.6 (3-8)	27.8 (18.6-36.6)	641 (400-880)	-	-	932 (700-1110)
* E. emiliopugini *	Formas (1989)§§	Puntra	2	-	203 (132-250)	25.45 (17-34)	85-633	1132 (500-2000)
* E. emiliopugini *	Penna and Solís (1999)	La Picada	1?	-	255 (181-314)	-	-	1062 (636-1459)
* E. emiliopugini *	Penna et al. (2005)	La Picada	1-2	-	255 (177-342)||	-	-	1053 (723-1401)
* E. emiliopugini *	Nuñez (2003)¶¶	not specified	2	-	640 (400-880)	-	-	507-1320

†Authors indicated that the second or third harmonic is dominant in *E.roseus*, *E.septentrionalis* and *E.calcaratus*, so here we reported the high frequency of the third harmonic for these three species. ‡Formas (1985) gave two inferior limits for this range: 1900 in the text, and 1100 in his Table 1. §These values were extracted from Table 2 of Nuñez et al. (2012a), who cited as source to Formas (1985) (where these values do not appear). |Formas (1985) gave two inferior limits for this range: 2700 in the text, and 3300 in his Table 1. ¶Formas and Brieva (1994) gave a different range for this species (6-7), citing Formas (1985). #As *E.queulensis*. ††These values were extracted from the text of Nuñez et al. (2012a), but in their Table 2 appear different range limits. ‡‡As *E.vittatus*. §§The values of the parameters were extracted from his Table 2, except the range of fundamental frequencies, which appears in the text; in the text there is also a different mean of pulses per note (27) and a different range of dominant frequencies (729-1320). ||CL of the single-note call. ¶¶Nuñez (2003) compiled the N/C, CL and range of DF for the eight species known at that time, but their values differ in some cases from the cited sources; here only included the values of *E.emiliopugini* reported in his Table 4, whose values of CL and DF are different of the original source (Formas 1989).

Immunological, allozyme and RFLPs studies

Since the mid-1970s, several immunological techniques and enzymatic systems (e.g., lactate dehydrogenases, hepatic hexokinases) were used to solve taxonomic and systematic problems of the anurans of the temperate forests of South America, including the genus *Eupsophus*. However, the earliest studies with enzymes (Díaz and Veloso 1979, Díaz 1981, 1986) had a more systematic orientation at the genus level and included only a few species of *Eupsophus*. Here we consider only those molecular studies focused on estimating genetic differentiation and relationships among the species of the genus. Similarly to morphometric analyses, allozyme studies revealed greater genetic differentiation between species groups (Formas et al. 1983) than within groups (Formas et al. 1983; Díaz 1986; Formas et al. 1992; Formas 1993; Ibarra-Vidal et al. 2004). In fact, some species such as *E.roseus* and *E.migueli* (Díaz 1986), and *E.contulmoensis* and *E.nahuelbutensis* (Ibarra-Vidal et al. 2004) are almost genetically indistinguishable according to this technique. The comparative studies of morphometry and allozymes showed that in general there is more disagreement (Formas et al. 1983; Formas et al. 1991, within *E.roseus*; Formas et al. 1992) than concordance (Formas 1993) between the morphological and genetic differentiation within the genus. Ibarra-Vidal et al. (2004) was the last study in which these markers were used in the genus, where two diagnostic loci between *E.septentrionalis* and *E.roseus* (among 19 putative loci), and less differentiation between *E.septentrionalis* and its geographically closest congeners, *E.contulmoensis* and *E.nahuelbutensis*, were reported. These allozyme patterns, particularly the almost fixed differences between *E.septentrionalis* and *E.roseus*, were used to support the specific status of *E.septentrionalis* (Ibarra-Vidal et al. 2004; Table 1). Only one study investigated intraspecific genetic variation using these markers: Formas et al. (1991) analyzed the allozyme variation among seven populations of *E.roseus*, representing a substantial part of its distribution. These authors found low levels of genetic differentiation among populations and interpreted that in support of its taxonomic status. It should be noted that in that study, the population of P.N. Nahuelbuta (type locality of *E.nahuelbutensis*; Ortiz and Ibarra-Vidal 1992) was included as part of *E.roseus*. The only immunological study focused exclusively on the relationships of the genus *Eupsophus* was Formas and Brieva (1992), who used precipitin tests in agar-gel. Although the focus of that study was mainly to examine the relationships of *Eupsophus* with other genera, they found a great affinity among some species of the *roseus* group and ratified the differentiation of the genus into two groups previously observed with chromosomal (Formas 1991) and bioacoustic (Formas 1985) evidence. Regarding RFLP markers, a single taxonomic study (Nuñez et al. 1999) used this technique to distinguish between the morphologically similar species *E.calcaratus* and *E.roseus*. They found identical restriction patterns of mitochondrial DNA within each species (two localities each) using two restriction enzymes.

Studies with DNA sequences

**Figure 2.**
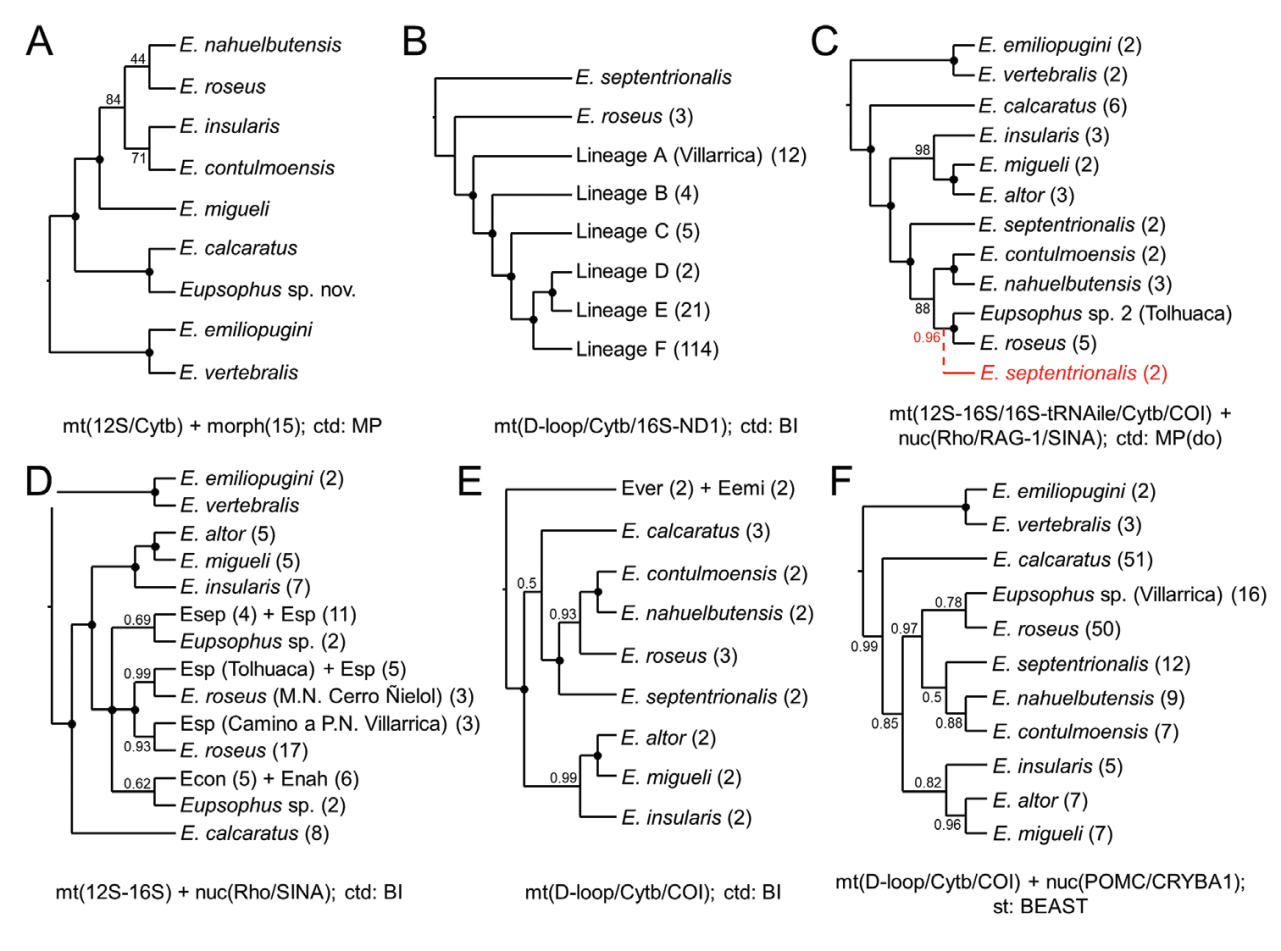
Phylogenetic hypotheses of *Eupsophus* obtained with DNA sequences. In some of these studies several phylogenetic analyses were made but here we show the hypotheses preferred by the authors. The trees were simplified by merging the terminal nodes by species or other relevant groupings and uniforming the branch lengths, but maintaining the original topologies. The numbers next to the nodes indicate the bootstrap or jackknife support values for the maximum parsimony (MP) analyses or posterior probability for those of Bayesian inference (BI). Black circles over the nodes indicate maximum support. The number of specimens included for each taxon or population is indicated in parentheses (omitted when only one was included). When relevant, the localities of origin of some specimens are indicated in parentheses. For simplicity, some names were abbreviated (for example, Esep = *E.septentrionalis*; Esp = *Eupsophus* sp.). Below the trees are indicated the gene fragments used, whether they are mitochondrial (mt) or nuclear (nuc), the analysis strategy (concatenated: ctd; species tree: st) and the phylogenetic reconstruction method used. **A**Nuñez (2003); this is the only tree of those shown where morphological characters (15) were included to build it **B**Nuñez et al. (2011); the only one of these studies where not all species of the genus were included; lineages A-F were considered a priori as *E.calcaratus***C**Blotto et al. (2013); the alternative position of *E.septentrionalis* (with its respective support value) obtained with a Bayesian analysis of the same data set is shown in red; the method used was MP with direct optimization (do); the support values correspond to jackknife absolute frequencies **D**Correa et al. (2017); note that several undescribed populations (*Eupsophus* sp. = Esp) appear intermixed with some nominal species of the *roseus* group; in this analysis *E.contulmoensis* (Econ) and *E.nahuelbutensis* (Enah) make up a clade but they are not reciprocally monophyletic **E**Suárez-Villota et al. (2018a); in this analysis *E.vertebralis* (Ever) and *E.emiliopugini* (Eemi) are not reciprocally monophyletic **F**Suárez-Villota et al. (2018b); they obtained a different topology within the *roseus* group in maximum likelihood and BI analyses of the same concatenated data set (not shown).

These studies have aimed to estimate the phylogenetic relationships within *Eupsophus*, its phylogenetic position with respect to other anuran groups, the phylogeographic history of one of its species (*E.calcaratus*) and its species diversity with species delimitation approaches (Fig. 2). Nuñez (2003) was the first study in which DNA sequences were incorporated to investigate the phylogenetic relationships of the genus. Nuñez (2003) included only one specimen per species (eight), obtaining a high support for the monophyly of the genus and its division into two groups, with *E.calcaratus* as sister of the rest of the species of the *roseus* group (Fig. 2A). Two later studies including more than one species (but still only one specimen of each) defined the phylogenetic position of the genus with respect to other anuran taxa. Correa et al. (2006), although including only five species of the genus, obtained a topology within *Eupsophus* congruent with that of Nuñez (2003) and found a close relationship of this genus with *Alsodes*, while Pyron and Wiens (2011) also recovered a well-supported sister relationship between *Eupsophus* and *Alsodes*, but with specimens wrongly labeled as *Batrachyla* and *Hylorina* nested within a monophyletic *Eupsophus* (confusion clarified by Blotto et al. 2013). Subsequent studies have included more than one specimen per species, so they have also allowed to assess the phylogenetic relationships among populations. Nuñez et al. (2011) reconstructed the phylogeographic history of *E.calcaratus* with mitochondrial sequences, including samples of most of its distribution range. They considered the six main groups identified in their phylogenetic analyses (labeled A to F) as “diagnostic of species lineages” (Fig. 2B), highlighting the great divergence between lineage A (locality of Villarrica) and the rest of the lineages (which they recovered as the sister taxon to *E.calcaratus*; see comment below). Nuñez et al. (2012a), in the description of *E.altor*, performed a phylogenetic analysis with a fragment of the control region (including samples of *E.calcaratus*, *E.roseus* and *E.migueli*), in which a sister relationship between *E.altor* (samples only from the type locality) and *E.migueli* was recovered (not included in Fig. 2). They included the molecular divergence between both species in the diagnosis of *E.altor* (nine nucleotide substitutions, according to the paper), but an examination of the sequences of Nuñez et al. (2012a) shows that this figure is higher (22 sites with fixed differences between both species and seven additional variable sites within *E.altor*; see comment in Correa et al. 2017). Blotto et al. (2013) performed a phylogenetic analysis of *Eupsophus* and *Alsodes* with mitochondrial and nuclear genes, including the 11 nominal species of *Eupsophus* recognized at that time, and in some cases more than one locality per species (Fig. 2C). They recovered the two species groups and ten of the eleven species as well-supported lineages, except for *E.queulensis* and *E.septentrionalis*, which were sympatric and had an extremely low sequence divergence (and consequently they were synonymized). Blotto et al. (2013) also suggested that one specimen from Tolhuaca probably represents an undescribed taxon, sister to *E.roseus* (Fig. 1). Correa et al. (2017) reassessed the species diversity of *Eupsophus*, specifically of the *roseus* group (see the next section), and estimated the phylogenetic relationships within the genus, using mitochondrial and nuclear sequences and including a greater number of specimens and localities than Blotto et al. (2013). Correa et al. (2017) found support for both species groups and for a topology within the *roseus* group consistent with that of Blotto et al. (2013) (although reduced to only four species; Fig. 2D). Suárez-Villota et al. (2018a) used a novel combination of mitochondrial sequences for reconstructing the relationships within the genus with a few specimens per species, but following the same taxonomy of Blotto et al. (2013). They obtained a high support for both species groups and recovered *E.calcaratus* in a different position with respect to previous studies (Nuñez 2003, Blotto et al. 2013, Correa et al. 2017; Fig. 2E). More recently, Suárez-Villota et al. (2018b) used a set of mitochondrial and nuclear genes and several phylogenetic approaches to reconstruct the relationships within the genus and estimate its species diversity with species delimitation approaches (see next section). They included an even greater number of specimens than Correa et al. (2017) (although a similar number of localities), obtaining a strong support for the species groups, but different positions for *E.calcaratus* depending on the analysis: the same position as in the hypothesis of Suárez-Villota et al. (2018a) (in a maximum likelihood analysis with concatenated sequences) or as the sister species of all the other species of the *roseus* group (in their species tree analyses). They also obtained a weak support for an alternative position of *E.septentrionalis*, which is congruent with previous hypothesis (Blotto et al. 2013, Suárez-Villota et al. 2018a), and strong support for recognizing the Villarrica lineage as a new putative species, although as the sister taxon to *E.roseus* (differing from the position found by Nuñez et al. 2011). Furthermore, Suárez-Villota et al. (2018b) estimated diversification times within the genus, finding that their delimited species diverged from 0.396 to 0.023 Mya (means). In summary, the relationships among the most of nominal species of the *roseus* group are well-supported by several studies (the clades *E.insularis* + (*E.migueli* + *E.altor*) and *E.contulmoensis* + *E.nahuelbutensis*, the position of *E.calcaratus* as sister taxon of all the other species of the *roseus* group), with the notable exception of *E.septentrionalis*, whose position fluctuates between studies (e.g., Blotto et al. 2013, Suárez-Villota et al. 2018a, b). Also, the position of the two putative species with respect to *E.roseus* (Villarrica and Tolhuaca populations) is uncertain, since both have not been included simultaneously in any study (Correa et al. 2017 included specimens from the surroundings of Villarrica, but not from the exact location where the new species would be found). Finally, a series of populations included by Correa et al. (2017) (*Eupsophus* sp. = Esp of Fig. 2D), whose geographic and phylogenetic position is intermediate with respect to *E.roseus*, *E.septentrionalis*, *E.contulmoensis* and *E.nahuelbutensis*, currently cannot be assigned to any of these species since they were not included in the species delimitation analyses of Suárez-Villota et al. (2018b). With respect to the two species of the *vertebralis* group, they show a very low degree of genetic divergence and are not always recovered as reciprocally monophyletic groups (Suárez-Villota et al. 2018a) or with high support values (Blotto et al. 2013). This low degree of divergence is reflected in the estimated time of separation of both species, which is the lowest in the genus (mean of 23 kya).

#### Species delimitation studies

Recently, two studies have focused explicitly on the delimitation of species, particularly in the *roseus* group (Correa et al. 2017, Suárez-Villota et al. 2018b). These two studies present contrasting views of the diversity of the genus (six and eleven species, respectively), so it is pertinent to review the evidence and methodology that supports both proposals, and their taxonomic and biogeographic implications. Correa et al. (2017) used one mitochondrial and two nuclear fragments of relatively conserved genes to reassess the species diversity of the *roseus* group, applying three unilocus species delimitation approaches. The sampled populations, many of them not described, cover the whole distribution of the genus, but are concentrated between 36 and 40°S, where the greatest diversity of species of the *roseus* group is found. In addition, they reviewed the chromosomal and bioacoustic evidence of the genus, which was used to choose between different delimitation scenarios. The proposal of Correa et al. (2017) represents a novel view of the diversity of species of the genus, recognizing only four species in the *roseus* group (Fig. 1). The proposed synonymizations were also supported by non-molecular arguments. Biogeographically, these changes imply a more simplified scenario since three of the synonymized species (*E.contulmoensis*, *E.nahuelbutensis* and *E.altor*) had distributions surrounded by populations of other species according to literature records. On the other hand, Suárez-Villota et al. (2018b) used three mitochondrial fragments (more variable) and two nuclear regions analyzed with several unilocus and multilocus species delimitation methods. The number of samples was double, but the number of localities was roughly the same as that of Correa et al. (2017). Their sampling scheme also covered the entire distribution range of the genus, but most of sampled populations are located between 39 and 46°S (and half of the localities included belong to *E.calcaratus*). Although Suárez-Villota et al. (2018b) used more sophisticated methods (multilocus), making use of mitochondrial and nuclear sequences, they did not explicitly consider non-molecular evidence to support their proposal. From a taxonomic point of view, Suárez-Villota et al. (2018b) reverted the changes proposed by Correa et al. (2017), returning to the previous classification of ten species, to which a new one not described would be added (Fig. 1). Biogeographically, this proposal implies that several species of the *roseus* group have restricted distributions, maintaining the same pattern of overlap between some species that is derived from the accumulated information of the literature (see fig 2 of Correa et al. 2017, and the collection of localities below).

#### Genomic studies

The recent description of the mitochondrial genomes of two species (*E.vertebralis* and *E.emiliopugini*) (Suárez-Villota et al. 2018a) marks the beginning of the genomic studies in the genus. Both species exhibit the same mitochondrial gene order as other neobatrachian frogs, and their mitogenomes are composed by 13 protein-coding genes, two ribosomal RNA genes, 22 transfer RNA genes, and a non-coding control region. Both genomes share 94.5% identity, which agrees with the low genetic divergence observed between the two species in several phylogenetic studies (e.g., Blotto et al. 2013, Correa et al. 2017, Suárez-Villota et al. 2018b).

#### Geographic distributions

**Figure 3.**
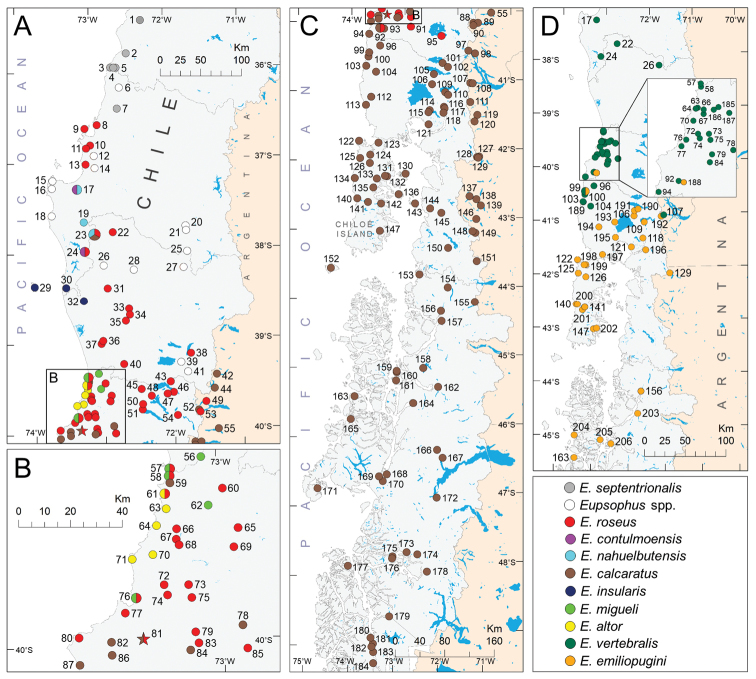
Compilation of localities of Eupsophus species gathered from the literature (see the complete list of localities in Appendix 1). Multicolored circles and the star indicate localities where two or three species of the same group have been reported in the same or different sources. White circles indicate the localities where two undescribed species have been identified (Villarrica and Tolhuaca), two undetermined populations included in this study (Fig. 4) and several ones considered by Correa et al. (2017) as E.roseus, whose taxonomic status is uncertain according to the current taxonomy (Suárez-Villota et al. 2018b). Thin gray lines within Chile represent boundaries of Administrative Regions.

The genus is distributed approximately between 35°28'S (Núñez and Gálvez 2015) and 49°25'S (Asencio et al. 2009) in Chile, and between 39°20'S and 43°S in Argentina (Úbeda 2000, Vaira et al. 2012, Blotto et al. 2013) (Fig. 3). The distribution range of the *roseus* group is the same as that of the genus (Fig. 3A–C), but that of the *vertebralis* group is more restricted (37°19' to 45°30'S, approximately; Fig. 3D). The most recent sources of range maps of *Eupsophus* species are Nuñez (2003), Rabanal and Nuñez (2008), Correa et al. (2017) and IUCN (2019). Nuñez (2003) and Rabanal and Nuñez (2008) contain highly congruent maps of eight species (*E.roseus*, *E.calcaratus*, *E.insularis*, *E.vertebralis*, *E.migueli*, *E.contulmoensis*, *E.emiliopugini*, and *E.nahuelbutensis*) generated with point occurrences and areas, respectively. Correa et al. (2017) reviewed the geographic information of the genus and compiled literature records to define the distribution ranges of the ten species recognized until that date, with an emphasis on the *roseus* group and the Chilean portion of the distribution. However, their maps (their fig. 2) were only intended to represent the boundaries among species that can be inferred by combining all the occurrence points collected from the literature. Correa et al. (2017) showed that the eight species of the *roseus* group exhibited a high degree of overlap, including several cases of the presence of more than one species in the same locality reported in the same or different publications (see details in S4 File of Correa et al. 2017 and Appendix 1). These instances of sympatry were not considered in the previous reviews or map sources, where a general pattern of allopatry among species of the same group was assumed (e.g., Formas 1989, Formas and Brieva 1994, Nuñez et al. 1999). Recently, the IUCN (2019) updated the assessments of *Eupsophus* species, adopting the taxonomy of Correa et al. (2017) (six species, Fig. 1), so its maps (areas representing the extent of occurrence) incorporated the synonymizations proposed by those authors. Despite being the most recent, the maps of IUCN (2019) do not adequately reflect the distribution limits of some species according to the literature (see details below). Here we update and complement the compilation of localities made by Correa et al. (2017) (Fig. 3 and Appendix 1), considering the current taxonomy (ten nominal species plus several undescribed populations), and highlight the inconsistencies that arise when all the available geographic information of the genus is compared.

##### Eupsophus septentrionalis

Taxon classificationAnimaliaAnuraCycloramphidae

Fig. 3A

###### Type locality.

R.N. Los Queules (Ibarra-Vidal et al. 2004); the same of *E.queulensis* (Veloso et al. 2005); locality 4 of Fig. 3A.

###### Geographic distribution.

One of the six species of the *roseus* group considered endemic to Chile, which currently presents a restricted distribution according to Suárez-Villota et al. (2018b). Its distribution range covers a narrow strip of the Coastal Range between 35°28' and 36°27'S (Ibarra-Vidal et al. 2004, Núñez and Gálvez 2015). Here we included a record omitted by Correa et al. (2017) (locality 1 of Fig. 3 and Appendix 1) that extends its distribution range almost 45 km to the north (Núñez and Gálvez 2015). Currently, this record constitutes the northern limit of the genus, which was incorporated in the new map of the IUCN (2019) as part of *E.roseus* (as well as all localities attributed to *E.septentrionalis*).

##### Eupsophus roseus

Taxon classificationAnimaliaAnuraCycloramphidae

Fig. 3A–C

###### Type locality.

Valdivia (Cei 1962a, b); locality 72 of Fig. 3B.

###### Geographic distribution.

The distribution range of this species is the most difficult to define from the literature, because its distribution limits differ among sources and four species were described within its range in Chile (*E.migueli*, *E.contulmoensis*, *E.nahuelbutensis* and *E.altor*), without clarifying the level of sympatry between them. In fact, *E.roseus* has been recorded in the type localities of some of these species: M.N. Contulmo (Ortiz et al. 1989, although Nuñez 2003 discarded its presence there), P.N. Nahuelbuta (Nuñez et al. 1999) and Mehuín (Formas et al. 1980, Puga 1986, Méndez et al. 2005). The maps of Nuñez (2003) and Rabanal and Nuñez (2008) are not very useful either, because they do not coincide in the northern and southern limits and restrict this species only to Chile. According to Formas (1979) and Formas et al. (1991), its northern limit in Chile is Concepción City (36°50'S), but subsequent sources limit it to Nahuelbuta Range (approximately 37°50'S; Nuñez et al. 1999, Rabanal and Nuñez 2008) or further south (Tolhuaca, 38°13'S; Nuñez 2003), ignoring several older records (e.g., Tomé, Cei 1962a, 1962b, as *E.grayi*; Tumbes, Grandison 1961; Fig. 3A). In contrast, the map of the IUCN (2019) extends its northern limit to ~35°28'S, encompassing completely the distribution range of *E.septentrionalis* (see above), and includes the few confirmed localities from Argentina (see below). Also, this map covers completely the distribution ranges of *E.migueli* and *E.altor*, and the continental area where *E.insularis* has been recorded (see below). According to Nuñez et al. (1999), the southern limit of *E.roseus* in Chile would be the Calle-Calle River basin (approximately 39°50'S), from where would be replaced by *E.calcaratus* southwards. The map of the IUCN (2019) is concordant with this pattern of allopatry between these species, though there are literature records of both species that surpass that limit (reviewed by Correa et al. 2017; Fig. 3B, C). Here we added an old literature record that implies the presence of *E.roseus* further south, until Cordillera Pelada (Puga 1986; locality 92 of Fig. 3C). Correa et al. (2017) discovered, using molecular evidence, a locality where *E.roseus* and *E.calcaratus* coexist (Naguilán, ~40°S, locality 81 of Fig. 3B, represented by a star), which would be the only confirmed site where two species of the *roseus* group live in sympatry. More recently, Suárez-Villota et al. (2018b) extended the distribution range of *E.roseus* further south on the western foothills of Andes in Chile (Los Mañíos, ~40°20'S) and demonstrated that effectively there are populations of *E.calcaratus* north of some localities of *E.roseus*. Taken together, these last two studies show that both species are present in Chile between 39°55' and 40°20'S approximately, although the degree of sympatry between them is currently unknown. Until 1996 (see account of *E.calcaratus*), *E.roseus* was considered as the only species of the genus in Argentina (e.g., Cei 1980), but recently its presence in that country has been debated (e.g., Vaira et al. 2012), where some populations have been unsteadily assigned to *E.roseus* and/or to *E.calcaratus* (discussed in Blotto et al. 2013). Blotto et al. (2013) confirmed the presence of *E.roseus* in that country (around 39°50'S, Fig. 3A), which suggests that the populations of Argentina north of that latitude, which were previously considered as *E.calcaratus* (Úbeda 2000), might correspond to *E.roseus*. Moreover, the finding of *E.roseus* in Los Mañíos (see above) shows that this species reaches further south through the Chilean Andes, which suggests the need to reevaluate the taxonomic status of the populations located in Argentina at the same latitude.

##### Eupsophus nahuelbutensis

Taxon classificationAnimaliaAnuraCycloramphidae

Fig. 3A

###### Type locality.

P.N. Nahuelbuta (Ortiz and Ibarra-Vidal 1992); locality 23 of Fig. 3A.

###### Geographic distribution.

Another of the six species of the *roseus* group endemic to Chile, which would have a restricted distribution according to Suárez-Villota et al. (2018b). Together with *E.contulmoensis*, they are the two species of the genus endemic to the Nahuelbuta Range. *Eupsophusnahuelbutensis* has been recorded in only two additional localities (Nuñez 2003): Ramadillas (where also *E.contulmoensis* was reported by Ortiz and Ibarra-Vidal 2005) and Rucapehuén. The map of Nuñez (2003) includes these three records, but that of Rabanal and Nuñez (2008) shows an area that exceeds the limits defined by those localities.

##### Eupsophus contulmoensis

Taxon classificationAnimaliaAnuraCycloramphidae

Fig. 3A

###### Type locality.

M.N. Contulmo (Ortiz et al. 1989); locality 24 of Fig. 3A.

###### Geographic distribution.

Another of the six species of the *roseus* group endemic to Chile, specifically to the Nahuelbuta Range, which would have a restricted distribution according to Suárez-Villota et al. (2018b). There are few records of this species in the literature (see Appendix 1). However, Ortiz and Ibarra-Vidal (2005) pointed out that this species has a wider distribution on the western slopes of the Nahuelbuta Range, between the south of the Biobío River (~37°10'S) and the latitude of the town of Tirúa (~38°20'S). On the other hand, the maps of Nuñez (2003) and Rabanal and Nuñez (2008) restrict the distribution of this species to its type locality and surroundings.

##### Eupsophus insularis

Taxon classificationAnimaliaAnuraCycloramphidae

Fig. 3A

###### Type locality.

Isla Mocha (Philippi 1902, Formas and Vera 1982); locality 29 of Fig. 3A.

###### Geographic distribution.

Another of the six species of the *roseus* group endemic to Chile, which would have a restricted distribution according to Suárez-Villota et al. (2018b). Correa et al. (2017) reported its presence in two localities on the southern part of the Nahuelbuta Range, one of them in front of Isla Mocha (Primer Agua), which were not included in the species delimitation study of Suárez-Villota et al. (2018b). We recognize these populations as *E.insularis* because of their close phylogenetic relationship with specimens from Isla Mocha and because they clearly belong to a clade other than the one that includes the geographically closest species (*E.contulmoensis*, *E.nahuelbutensis* and *E.roseus*; Correa et al. 2017). The map of IUCN (2019) coincides with previous representations (Nuñez 2003, Rabanal and Nuñez 2008) that restrict the species only to Isla Mocha. However, the continental populations assigned to this species by Correa et al. (2017) would be within the distribution range of *E.roseus* according to IUCN (2019).

##### Eupsophus migueli

Taxon classificationAnimaliaAnuraCycloramphidae

Fig. 3B

###### Type locality.

Mehuín (Formas 1978a); locality 58 of Fig. 3B.

###### Geographic distribution.

Another of the six species of the *roseus* group endemic to Chile, restricted to a narrow coastal strip between 39°23' and 39°51'S (Fig. 3B). *Eupsophusmigueli* was described from two coastal localities in Chile, Mehuín and Los Molinos (39°25' to 39°51'S; Formas 1978a), but later its distribution was expanded eastward to a few nearby localities, like San José de la Mariquina (Méndez et al. 2005) and Colegual Alto (Nuñez et al. 2012a) (Fig. 3B). Cumulative literature records imply the sympatry of *E.migueli* and *E.roseus* at Mehuín, Queule and Los Molinos (Appendix 1 and Fig. 3B). Available maps restrict its distribution to its type locality and surroundings (Nuñez 2003, Rabanal and Nuñez 2008), ignoring the other locality of the original description, Los Molinos. The map of IUCN (2019), by including the entire range of *E.altor*, extends the distribution of *E.migueli* further south, but it does not include Los Molinos either. To the north, this map surpasses the northernmost record of the species by about 20 km, but does not include the locality of San José de la Mariquina, which extends its distribution significantly to the east (compare with the map of Correa et al. 2017). Moreover, the map of *E.roseus* of the IUCN (2019) implies that both species are completely sympatric across the entire distribution range of *E.migueli*.

##### Eupsophus altor

Taxon classificationAnimaliaAnuraCycloramphidae

Fig. 3B

###### Type locality.

Parque Oncol (Nuñez et al. 2012a); locality 70 of Fig. 3B.

###### Geographic distribution.

Another of the six species of the *roseus* group endemic to Chile, which presents a restricted distribution according to Suárez-Villota et al. (2018b). *Eupsophusaltor* was reported originally from four localities (39°29' to 39°42'S, Nuñez et al. 2012a), but a map by Nuñez et al. (2012b) shows six points without mentioning the localities (not included in Fig. 3B). In any case, all these localities are between the two original ones of *E.migueli*, Mehuín and Los Molinos (localities 58 and 76 of Fig. 3B). In one of the original localities, Alepúe, *E.roseus* has also been recorded (Blotto et al. 2013). This last record can be added to the others mentioned above, which indicate the presence of *E.roseus* in several coastal locations where *E.migueli* and *E.altor* are found, but the map of the IUCN (2019) shows a continuous distribution of *E.roseus* that completely covers those of both species.

##### Eupsophus calcaratus

Taxon classificationAnimaliaAnuraCycloramphidae

Fig. 3A–C

###### Type locality.

Chiloé Island (locality not specified; Günther 1881, Formas and Vera 1982); localities 122-126, 131-135, 140-142 and 147 of Fig. 3C.

###### Geographic distribution.

This is the species with the widest distribution of the genus, slightly surpassing the 49°20'S toward the south (Fig. 3C). However, its northern limit cannot be clearly defined from the literature since there are three records north of the Calle-Calle River basin, the limit defined by Nuñez et al. (1999) (around 39°50'S): P.N. Nahuelbuta (locality 23 of Fig. 3A), Villarrica (39) and Mississipi (59). Its presence in P.N. Nahuelbuta (Ortiz and Ibarra-Vidal 1992; Fig. 3A) was questioned by Nuñez (2003) and the inclusion of the populations around Villarrica in this taxon was challenged by Nuñez et al. (2011), Correa et al. (2017) and Suárez-Villota et al. (2018b). Thus, the record of the species in Mississipi would remain, but this population would be entirely surrounded by populations of *E.migueli* and *E.roseus* according to all the available information. The populations near Reumén (39°57'S), recently reported by Suárez-Villota et al. (2018b), would also be surrounded by populations of *E.roseus*, but in this case these findings are supported by molecular evidence. Together with Naguilán (locality 81, where *E.roseus* also is present, Correa et al. 2017) these localities constitute the northern limit confirmed by molecular phylogenetic analyses. All these findings do not coincide with the limits that appear on the maps of IUCN (2019), where *E.calcaratus* is replaced to the north by *E.roseus* around 40°S in Chile. In Argentina, the presence of this species was first reported by Christie and Úbeda (1996), but later, all the populations of the *roseus* group in that country were considered as *E.calcaratus* (39°34' to 43°S; Úbeda 2000; see comment in Vaira et al. 2012). However, the phylogenetic analyses of Blotto et al. (2013) (ratified by Correa et al. 2017) imply that two localities in Argentina correspond to *E.roseus* (Fig. 3A), which would be flanked to the north and south by populations of *E.calcaratus*. The maps of Rabanal and Nuñez (2008) and IUCN (2019) show that *E.calcaratus* reaches further north on the Argentine side, assuming that all the populations included in Úbeda (2000) and others that extend their distribution about 30 km further north belong to this species.

##### Eupsophus vertebralis

Taxon classificationAnimaliaAnuraCycloramphidae

Fig. 3D

###### Type locality.

Valdivia (Grandison 1961); locality 72 of Fig. 3D.

###### Geographic distribution.

It is known mainly in the coastal zone of Chile, between the north of the Nahuelbuta Range (37°19'S) and the Osorno coast (40°49'S). Only two localities outside this area are known, Tolhuaca (locality 26), on the western margin of the Andes, and Puerto Blest in Argentina (107; Basso and Úbeda 1999, Úbeda and Basso 2012a), on the other side of the Andes. However, this last point is closer to the records of *E.emiliopugini*. *Eupsophusvertebralis* and *E.emiliopugini* would have allopatric distributions according to Formas (1989) and Nuñez (2003), but two relatively recent records of *E.emiliopugini* (Raulintal and Pucatrihue, Olivares et al. 2014 and Suárez-Villota et al. 2018b, respectively; Fig. 3D) imply the sympatry of both species in the southern end of the distribution of *E.vertebralis*. The maps of Rabanal and Nuñez (2008) and IUCN (2019) also imply sympatry areas in Chile, but in different zones: on the Coastal Range according to Rabanal and Nuñez (2008) and on the western foothills of the Andes according to IUCN (2019). None of those sympatry areas is supported by the review of the literature records (Fig. 3D).

##### Eupsophus emiliopugini

Taxon classificationAnimaliaAnuraCycloramphidae

Fig. 3D

###### Type locality.

La Picada (Formas 1989); locality 106 of Fig. 3D.

###### Geographic distribution.

*Eupsophusemiliopugini* would be distributed both on the coast and the Andean zone, mainly in Chile, between 40°11' and 45°30'S, although it would be in sympatry with *E.vertebralis* in a small area of the Chilean Coastal Range (see above). In Argentina, it is present on the northwest and southwest coasts of Lago Puelo (Úbeda and Basso 2012b), where Arroyo Melo (Úbeda et al. 1999; locality 129) is located.

##### Eupsophus spp.

Taxon classificationAnimaliaAnuraCycloramphidae

Fig. 3A

###### Geographic distribution.

The two undescribed species mentioned in the recent literature (Fig. 1) are known from one locality each: Tolhuaca (*Eupsophus* sp. 2 of Blotto et al. 2013) and Villarrica (*Eupsophus* sp. of Suárez-Villota et al. 2018b) (both considered as *E.roseus* by Correa et al. 2017). Also, a series of populations located between 36°10' and 38°15'S, assigned to *E.roseus* by Correa et al. (2017), should be included here since they occupy intermediate phylogenetic and geographic positions among the species recognized by Suárez-Villota et al. (2018b). Almost all these localities are within the latitudinal limits defined for *E.roseus* according to historical records (see above), but as Correa et al. (2017) indicated, these populations cannot identify unambiguously to species level by their external characters. Other southernmost undetermined populations included in Correa et al. (2017) (Santa Amelia, Pumalal, Puringue and Malalhue) are considered here as *E.roseus* because they make up a well-supported monophyletic group with specimens from the type locality of that species (where the specimen from Naguilán is also included). The two new localities where phenotypic observations were done for this review (see below) are also included here.

#### Phenotypic observations

**Figure 4.**
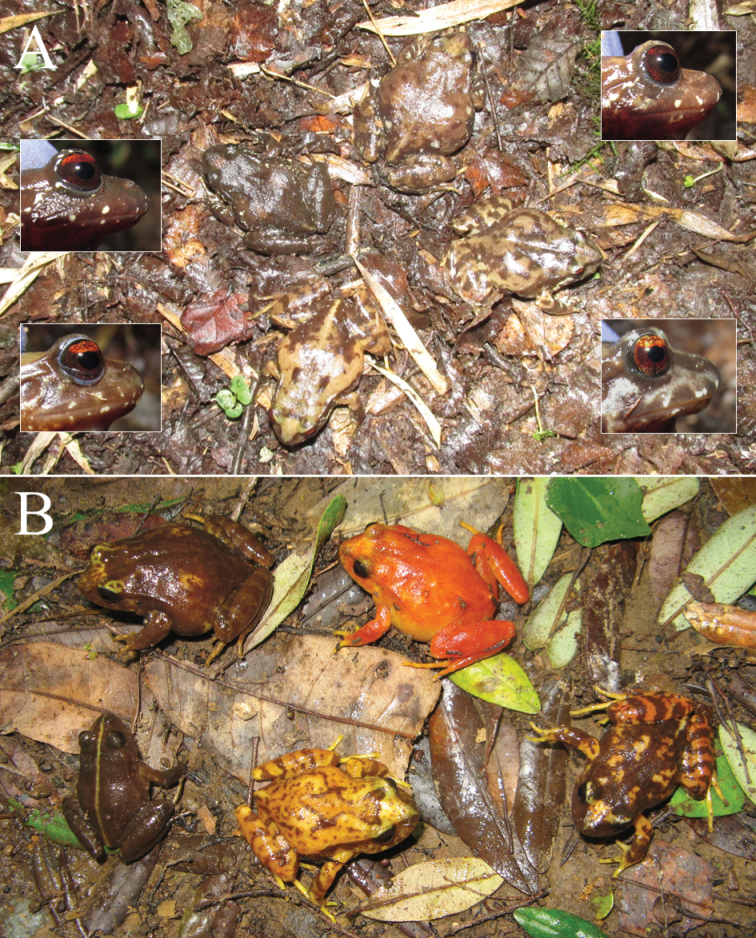
Cryptic coloration and variation of coloration patterns in two undetermined populations of the *Eupsophusroseus* group **A** adult females from Pidenco, showing cryptic coloration resembling the forest ground; insets show head profiles of the same individuals **B** adults and juveniles from Las Lianas exemplifying variation in coloration patterns. Both localities were included as *Eupsophus* sp. in the map of Fig. 3.

**Figure 5.**
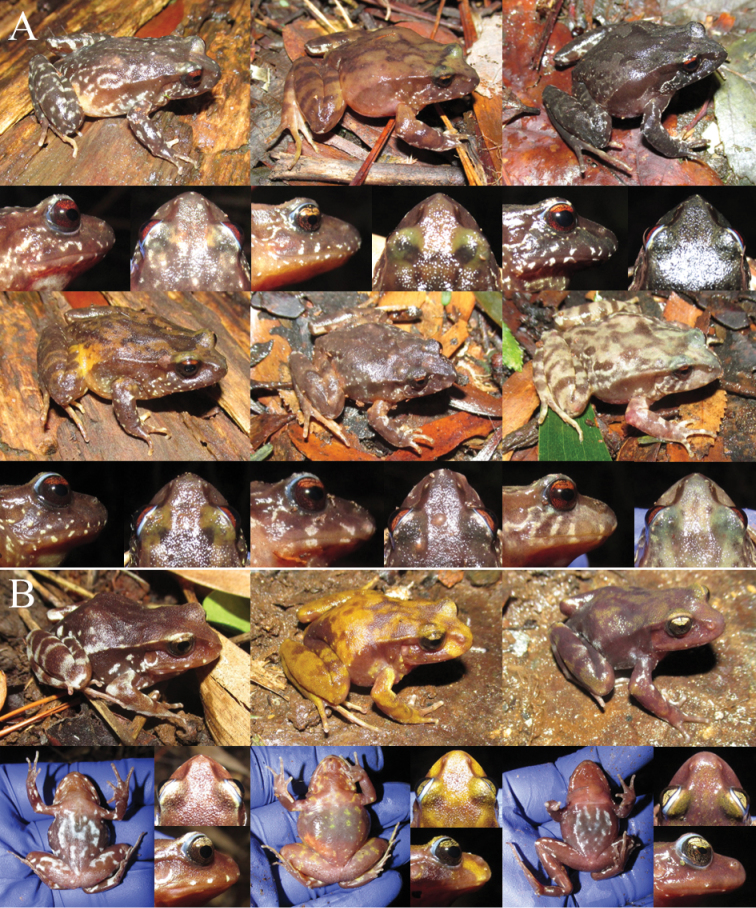
Examples of intrapopulation external variation in adult specimens of the type localities of two species of the *Eupsophusroseus* group **A***Eupsophusroseus* from Valdivia **B***Eupsophusmigueli* from Mehuín. Both examples illustrate the variation in dorsal and ventral (**B**) coloration, iris color and snout shape.

One of the contributions of Correa et al. (2017) was the explicit recognition of the high level of intrapopulation variation in external characters considered diagnostic in the taxonomy of the genus. Here we show additional examples of intrapopulation variation in the three external characters most frequently included in the diagnoses of *Eupsophus* species (dorsal and ventral color patterns, iris color, and lateral and dorsal snout profile; Table 1; see also Correa et al. 2017), in live animals of two undescribed populations (Fig. 4) and two type localities (Fig. 5). Figure 4 illustrates the variation in dorsal coloration patterns in specimens from Pidenco (A, four adults randomly selected, from a total of 13, to show also the typical cryptic coloration of the genus and the variation of iris color and snout profile) and Las Lianas (B, five specimens chosen among 19 to represent contrasting dorsal coloration patterns, including one with a thin vertebral line). Most of specimens from Las Lianas had uniform brown eyes and only one had the upper part of the iris yellowish. Moreover, the length and profile of the snout varied among these specimens (data not shown). Figure 4 shows the variation of body coloration patterns (dorsal and ventral), iris coloration and shape of snout (both in dorsal and lateral profile) in the type localities of *E.roseus* (A, Valdivia, where it is the only species of the *roseus* group that has been reported; see Fig. 3) and *E.migueli* (B, Mehuín, where also *E.roseus* would be present, see above and Fig. 3). The six specimens of *E.roseus* were selected from 16, collected in two sessions, in order to exemplify the variation of iris color, which ranges from reddish to pale orange, and shape of the snout, which varies in length and form in lateral and dorsal profile. The three specimens of *E.migueli* (Fig. 5B) were collected in two sessions (14 in total) and differ notably in dorsal and ventral coloration patterns and in snout profile. They also differ in coloration from the holotype, which had the dorsum grayish with two dark paravertebral areas and a thin light vertebral line (Formas 1978a). At Mehuín, where *E.migueli* and *E.roseus* supposedly coexist (see above), no specimens with the iris orange like *E.roseus* were observed.

#### Phylogenetic analyses

**Figure 6.**
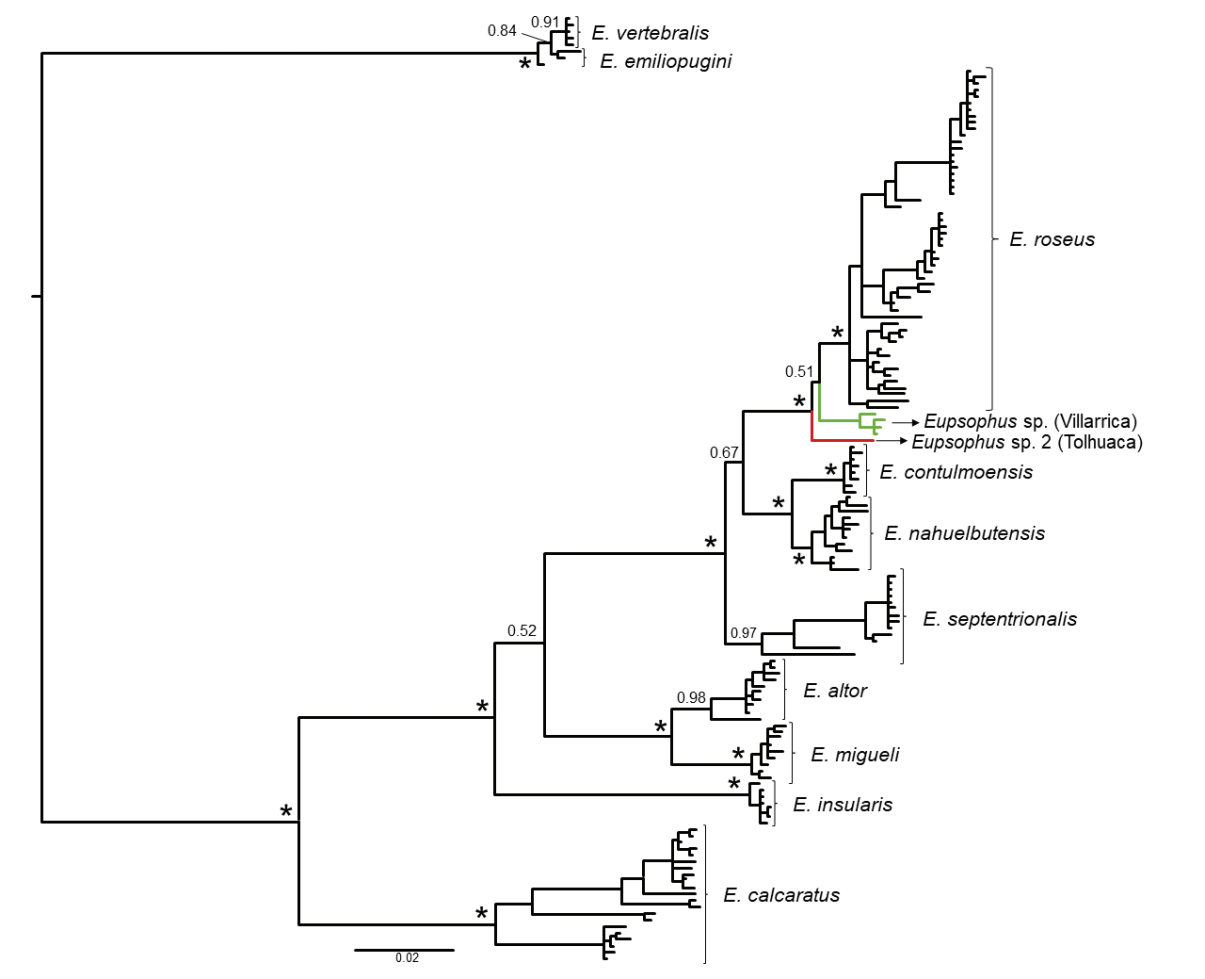
Consensus phylogram (50% mayority-rule) of the Bayesian analysis of the mitochondrial fragments cytochrome c oxidase subunit I and cytochrome b. For simplicity, the outgroup (Alsodesnorae) is not shown. Colored branches indicate the specimens of the two putative species: Villarrica (green) and Tolhuaca (red). The values next to the nodes are the posterior probabilities (pp); asterisks represent maximum values (pp = 1). Note that all species currently recognized (Suárez-Villota et al. 2018b) are supported by high pp values (> 0.97), except for both of the vertebralis group, wich are not reciprocally monophyletic. The scale bar under the tree represents the expected substitutions per site.

We obtained an alignment of 1304 nucleotide sites when the sequences of different length of both gene fragments were included (631 sites of cytb, 673 of COI), which was reduced to 998 when cutting ends with gaps (365 sites of cytb, 633 of COI). The four analyses (with or without sites with gaps, two or six partitions) recovered the two species groups and all the currently recognized nominal species of the *roseus* group as well-supported clades (posterior probability, pp > 0.97), but the topology within this group is variable among analyses, including some polytomies, and only partially congruent with previous phylogenetic studies (Fig. 2). Figure 6 shows the Bayesian consensus tree (15 002 sampled trees) of the analysis of the short alignment with six partitions. An important difference with respect to prior hypotheses is the position of *E.insularis* as the sister species of the all species of the *roseus* group, except for *E.calcaratus*; though in the analysis of the short alignment with two partitions appears as the sister species of *E.migueli* + *E.altor* like in previous studies. Another difference with respect to the most recent hypothesis (Fig. 2F) is the position of *E.septentrionalis*, recovered as the sister group of *E.roseus*, *E.contulmoensis*, *E.nahuelbutensis* and Villarrica and Tolhuaca populations, which is only consistent with the results of Suárez-Villota et al. (2018a) (Fig. 2E). However, *E.septentrionalis* also formed a polytomy with *E.roseus* + Villarrica + Tolhuaca and *E.contulmoensis* + *E.nahuelbutensis* clades in both analyses with two partitions. The four analyses showed the close relationship of Villarrica and Tolhuaca populations with *E.roseus*, all of which comprise a clade with maximal support. However, the reciprocal relationship between Villarrica and Tolhuaca populations could not be resolved since in three of the four analyses both putative taxa form a tritomy with *E.roseus* (Fig. 6 shows the only analysis where this relationship is resolved, but with low support). This lack of resolution could be due to the low number of variable nucleotide sites with respect to other studies where more genes were included, but in no case the Villarrica or Tolhuaca specimens appear mixed with those of *E.roseus*. Therefore, Tolhuaca population also should be considered a candidate species under the current taxonomy.

## Discussion

During the last six decades, the taxonomic and systematic research on ground frogs, beyond of species descriptions and estimations of phylogenetic relationships, has focused on solving three fundamental issues: the delimitation of the genus, its division into species groups and the estimation of its species diversity. The monophyly and distinction of *Eupsophus* with respect to its sister genus, *Alsodes*, is now well established based on morphological, chromosomal, bioacoustic, developmental and molecular phylogenetic evidence (Gallardo 1970, Lynch 1978, Nuñez 2003, Vera Candioti et al. 2011, Blotto et al. 2013). Likewise, the subdivision of the genus into two groups is supported by cumulative morphological, chromosomal, bioacoustic, genetic, immunological, and molecular phylogenetic evidence (see references in Results; reviewed in Nuñez 2003, although this author suggested that each group could represent a different genus). However, the number of species, which progressively increased from two (Lynch 1978) to a maximum of 11 (Nuñez et al. 2012a), decreased to six in the following five years (Blotto et al. 2013, Correa et al. 2017) and more recently, raised again to 11 (Suárez-Villota et al. 2018b; Fig. 1). This recent instability is due to two opposing views about the species diversity of the *roseus* group. Correa et al. (2017) used only unilocus species delimitation methods, but their proposal took into account the scarce chromosomal and bioacoustic differentiation within the group. Instead, Suárez-Villota et al. (2018b), using a bigger dataset and more sophisticated (multilocus) analyses, ratified the validity of the same nominal species recognized by 2013 and provided support for a new putative species. This last proposal implies the consolidation of the taxonomic work of the last decades and reinforces the idea that the species diversity of the genus could be underestimated (Nuñez et al. 2011, Blotto et al. 2013). Logically, this advance depends on the robustness of the previous taxonomy, but as shown in Correa et al. (2017) and here, there are enough precedents in the literature that allow to question the “traditional” taxonomy, something that was not considered by Suárez-Villota et al. (2018b). Most of these precedents were developed in Results, so below we only discuss the main problems that emerged from the comparison and critical analysis of all that information.

Diagnoses are fundamental in taxonomy, since diagnostic characters summarize the differences among closely related taxa (Winston 1999). However, we detected two general problems with the quality of diagnoses of *Eupsophus* species: the heterogeneity in the number and type of characters included and the use of very variable characters for distinguishing species of the same group. The heterogeneity can be clearly seen in Table 1 and implies that, over time, very different criteria have been applied to define which and how many characters are sufficient to diagnose the species. Indeed, only four characters have been included in four or more diagnoses (the first four characters of Table 1). Regarding character variation, Correa et al. (2017) showed, with examples from the literature and observations of live animals, that these same four characters vary intraspecifically. In fact, body coloration patterns, which are included in most diagnoses, vary even in the type series (Correa et al. 2017). These observations of the type material have been corroborated with examples of live specimens from the type localities of *E.roseus* and *E.altor* (Correa et al. 2017), and *E.roseus* and *E.migueli* (this study). These and additional examples from other populations show that variation in body coloration is widespread in the genus, but this phenomenon has rarely been recognized in the literature (Cei 1962a, 1962b, Nuñez 2003, Nuñez et al. 2012a) and its implications for the taxonomy never have been addressed. The other two external characters, iris color and snout shape (Correa et al. 2017; this study), and the shape of the xiphisternum (Díaz 1986) also vary extensively within species. Taken together, all this information weakens the evidence used to distinguish some species, particularly those whose diagnoses rest almost exclusively on these characters (e.g., *E.insularis* and *E.migueli*). These high levels of variation in diagnostic characters have deep consequences for the current taxonomy (Suárez-Villota et al. 2018b), since that proposal is based on material only from the type locality for several species and according to its proponents is concordant with the taxonomic work of the last decades.

Our review of the literature showed that, apart from external and internal morphology, morphometrics, karyotypes, and calls have been the main lines of evidence applied to the taxonomy and systematics of *Eupsophus*. Although these kinds of data have been rarely incorporated into diagnoses, they have been included in the descriptions of several species (Formas 1978a, 1989, Veloso et al. 2005, Nuñez et al. 2012a). Each of those three lines of evidence support the distinction between the two species groups, though they have limited utility to differentiate species within groups. Except in the case of the two species of the *vertebralis* group, *E.vertebralis* and *E.emiliopugini*, which are clearly differentiated by their karyotypes and to a lesser extent by their advertisement calls (Formas 1989), few species of the genus can be differentiated with these data. In fact, none of the species of the *roseus* group can be distinguished by their advertisement calls, since all the parameters used to describe them overlap extensively and the descriptions of the calls of some species differ among studies (Correa et al. 2017). The karyotypic evidence deserves an additional commentary, since it has been explicitly (Formas 1978b) or implicitly (Veloso et al. 2005) assumed that species of this genus have characteristic karyotypes. The comparison of all published karyotypes shows that this is not the case and that different karyotypes were described for the same species and locality by different authors (*E.roseus*, *E.migueli*, and *E.vertebralis*), suggesting strongly observer biases (Correa et al. 2017). Even though these differences were real, the level of intrapopulation and intraspecific variation in chromosome morphology and position of secondary constrictions would be as high as the variation at interspecific level (see Table 2), so that this type of evidence would not be useful in the taxonomy of the *roseus* group.

The review of the geographic information also revealed difficulties in establishing the spatial boundaries of the species of the genus. Recently, Correa et al. (2017) compiled records of the literature (that we expand here), showing a high degree of overlap of distribution ranges and cases of sympatry among species of the same group that had not been recognized in previous studies and reviews (e.g., Nuñez 2003, Blotto et al. 2013). These compilations of records differ from the most recent published maps (Nuñez 2003, Rabanal and Nuñez 2008, IUCN 2019), which show mainly allopatric distributions for species of the same group and do not coincide with each other for some species. These discrepancies between available maps and the points collected are closely linked to the four species (*E.migueli*, *E.contulmoensis*, *E.nahuelbutensis* and *E.altor*) described within of the distribution range of *E.roseus*, whose limits and degree of sympatry have been never precisely established. The records compiled here also show an overlap between the distribution ranges of *E.roseus* and *E.calcaratus*, which is partially supported by molecular evidence but does not coincide with the previously established limits (e.g., Nuñez 2003). The proposal of Correa et al. (2017), by expanding the taxonomic limits of *E.roseus* and *E.migueli*, resulted in a considerable reduction in the levels of overlap of the distribution ranges, but the rebuttal of Suárez-Villota et al. (2018b) implicitly meant returning to the confusing situation derived of the geographic information of the literature. Moreover, they added one more factor of uncertainty when affirming that some species (*E.migueli*, *E.altor*, *E.contulmoensis*, *E.nahuelbutensis*, *Eupsophus* sp. and *E.septentrionalis*) have “restricted distributions”, which implies that the genus would have a highly fragmented distribution at present. This pattern is incompatible with the information available since there are historical records of *E.roseus* (see map of Fig. 3) and taxonomically undetermined populations (Correa et al. 2017) between the localities assigned to these species. Currently, it is not clear how these intermediate populations would fit into the taxonomic scheme of Suárez-Villota et al. (2018b). The problems to define the boundaries between species are not only limited to Chile, where the greatest diversity of species is found, but also extend to Argentina where the boundary between *E.roseus* and *E.calcaratus* is not clear.

This review summarizes six decades of taxonomy and systematic research on *Eupsophus* (partially reviewed by Correa et al. 2017), but unlike the last comprehensive review treating these topics (Nuñez 2003) the information from various sources is compared. Only this retrospective and comparative approach allowed to reveal the high degree of variation described in some morphological characters used for the descriptions and diagnoses, the lack of significant differentiation in morphometrics and advertisement calls, and the incongruences in the chromosomal evidence and geographic data (see also Correa et al. 2017). These patterns agree with the general decoupling between the morphological and phylogenetic differentiation implied for the last phylogenetic studies (Blotto et al. 2013, Correa et al. 2017, Suárez-Villota et al. 2018b), which had already been suggested by the comparative studies with allozymes and morphometry (Formas et al. 1983, Formas et al. 1991, Formas et al. 1992). Moreover, a practical issue emerged from this comparative synthesis. Since the levels of intra/interspecific morphological variation and divergence among species are high but poorly known, especially in the *roseus* group (regardless of the taxonomy adopted), field identification would be reliable only within the assumed distribution ranges and, as we have demonstrated, there has not been consensus about them. Therefore, inconsistent diagnoses, field misidentifications and misleading geographic data might be intimately linked, explaining most cases of sympatry and range overlap inferred from the compilation of localities. In turn, erroneous geographic data might influence the identification of atypical specimens, particularly in the distribution limits and unexplored zones. The problem of field misidentification is expected to persist under the most recent taxonomic arrangement (Suárez-Villota et al. 2018b) since that proposal is mainly based on material from the type localities or surroundings (except for *E.calcaratus*) and, as we pointed out above, the diagnoses of *Eupsophus* species are unreliable and their geographic boundaries are still poorly defined.

In this context, phylogenetic and species delimitation studies with DNA sequences have emerged as an independent and powerful way to reassess the taxonomy of *Eupsophus*. However, except for Correa et al. (2017), those studies (Nuñez et al. 2011, 2012a, Blotto et al. 2013, Suárez-Villota et al. 2018a, b) have progressively reinforced the previous taxonomic work, without questioning the bases that support it. In addition, they have installed the idea that diversity at the species level would be underestimated by identifying two candidate species (Villarrica and Tolhuaca populations). Apparently, these advances constitute the consolidation of decades of taxonomic research based on other types of evidence, but the critical examination of the taxonomic literature done here allows us to outline two issues that weaken this assertion. First, there is scarce morphometric, karyotypic and bioacoustic differentiation and a very high level of intrapopulation variation in some external and internal characters (e.g., shape of the head, body coloration, shape of the xiphisternum) in the *roseus* group (patterns already noted by Correa et al. 2017), which excludes them as reliable sources of characters to distinguish the species. Taken together, these types of characters, which support most of the descriptions and diagnoses of the species, suggest that the diversity of the genus at species level is not well described so it is not clear how a delimitation approach based exclusively on molecular evidence can ratify such taxonomic scheme. Second, the claim that most species of the *roseus* group have “restricted distributions” (see above) has important consequences for the biogeography and taxonomy of the genus. Historical records and intermediate undetermined populations show that this pattern of isolated species does not adequately reflect the distribution of the genus, but more importantly, some of these populations occupy intermediate phylogenetic positions between some narrow-range species of the *roseus* group, decreasing the genetic divergence among them (Correa et al. 2017). The latter implies that the populations that make up these species with restricted distributions do not represent well the overall phylogenetic diversity of the genus, so that this dimension of its diversity is not adequately reflected by the current taxonomy. Therefore, future taxonomic and systematic studies of *Eupsophus*, whether molecular or not, should take into account the incongruities between the patterns of molecular, morphological, bioacoustic and chromosomal divergence and incorporate more intermediate populations to obtain a more accurate estimate of its species diversity.

## Supplementary Material

XML Treatment for Eupsophus septentrionalis

XML Treatment for Eupsophus roseus

XML Treatment for Eupsophus nahuelbutensis

XML Treatment for Eupsophus contulmoensis

XML Treatment for Eupsophus insularis

XML Treatment for Eupsophus migueli

XML Treatment for Eupsophus altor

XML Treatment for Eupsophus calcaratus

XML Treatment for Eupsophus vertebralis

XML Treatment for Eupsophus emiliopugini

XML Treatment for Eupsophus

## Appendix 1. List of localities of *Eupsophus* species compiled from the literature

This list contains all localities included in Fig. 3, ordered by species, according to the current taxonomy (Suárez-Villota et al. 2018b; Fig. 1), and then by latitude, from north to south, or geographic proximity. Localities in bold indicate where more than one species of the same species group is present according to the literature (circles with two or three colors in Fig. 3) or according to the phylogenetic analysis of Correa et al. (2017) (sympatry of *E.calcaratus* and *E.roseus* in Naguilán, brown-red star of Fig. 3A–C). Under the category *Eupsophus* spp. we grouped some populations included in Correa et al. (2017) (whose taxonomic status currently is unclear), two undescribed populations included here, and two undescribed candidate species (Fig. 1).

*Eupsophusseptentrionalis* (Fig. 3A): 1) Estación Experimental Dr. Justo Pastor León, 2) R.N. Los Ruiles, 3) Trehualemu, 4) R.N. Los Queules, 5) 3 km east R.N. Los Queules, 7) Trehuaco.

*Eupsophusroseus* (Fig. 3A): 8) Tomé, 9) Tumbes, 10) Concepción (Cerro Caracol), 11) Laguna Grande (San Pedro), 13) Coronel, 22) Los Lleulles, 23) **P.N. Nahuelbuta**, 24) **M.N. Contulmo**, 31) 10 km west Galvarino, 33) Rucamanque, 34) M.N. Cerro Ñielol, 35) Maquehue, 36) Santa Amelia, 37) Pumalal, 38) Lago Tinquilco, 40) Cuesta Lastarria, 43) Pocura, 45) Malalhue, 46) Lago Pellaifa, 47) San Pablo de Tregua, 48) Panguipulli, 49) Lago Paimún (Argentina), 50) Fundo San Clemente, 51) Desembocadura del Lago Riñihue, 53) Termas de Epulafquén (Argentina), 54) Huilo Huilo; (Fig. 3B): 57) **Queule**, 58) **Mehuín**, 60) Puringue, 61) **Alepúe**, 65) Huifco (torre 21), 66) Iñipulli, 67) Bosque or Fundo San Martín, 68) Fundo Santa María, 69) Máfil (Torre 41), 72) Valdivia (city), 73) Cuesta de Soto, 74) Huachocopihue, 75) Llancahue, 76) **Los Molinos**, 77) Corral, 79) Camino Viejo a La Unión, 80) Reserva Costera Valdivia, 81) **Naguilán**, 83) Chamil, 85) Paillaco (Torre 140); (Fig. 3C): 91) Pichirropulli, 92) **Cerro Mirador (Cordillera Pelada)**, 95) Los Mañíos.

*Eupsophus* spp. (Fig. 3A): 6) Sector Guanaco or Cerro El Guanaco, 12) Cerros de Chiguayante, 14) Santa Juana, 15) Llico, 16) Quidico, 18) Las Lianas (this study), 20) Alto Biobío, 21) Loncopangue, 25) Pemehue, 26) Pidenco (this study), 27) Tolhuaca (*Eupsophus* sp. 2 of Blotto et al. 2013), 28) Río Traiguén, 39) Villarrica (*Eupsophus* sp. of Suárez-Villota et al. 2018b), 41) Camino a P.N. Villarrica.

*Eupsophusnahuelbutensis* (Fig. 3A): 17) **Ramadillas**, 19) Rucapehuén, 23) **P.N. Nahuelbuta**.

*Eupsophuscontulmoensis* (Fig. 3A): 17) **Ramadillas**, 24) **M.N. Contulmo**; also recorded at Reserva Forestal Contulmo, located 2.4 km SW, in a straight line, from M.N. Contulmo (not shown in Fig. 3).

*Eupsophusinsularis* (Fig. 3A): 29) Isla Mocha, 30) Primer Agua (Webb and Greer 1969 reported the presence of *E.roseus* at 7 km SSE Tirúa, the almost exact location of Primer Agua, so we left only this last record because it is supported by exact geographic information and molecular evidence), 32) Camino a Villa Las Araucarias.

*Eupsophusmigueli* (Fig. 3B): 56) Colehual Alto, 57) **Queule**, 58) **Mehuín**, 62) San José de la Mariquina, 76) **Los Molinos**.

*Eupsophusaltor* (Fig. 3B): 61) **Alepúe**, 63) Chanchán, 64) Llenehue, 70) Parque Oncol, 71) Curiñanco.

*Eupsophuscalcaratus* (Fig. 3A): 23) **P.N. Nahuelbuta**, 42) Lago Quillén (Argentina), 44) Lago Tromen (Argentina), 52) near Paso Carirriñe (Argentina), 55) Lago Lolog (Argentina); (Fig. 3B): 59) Mississipi, 78) Reumén (Suárez-Villota et al. 2018b included three very close localities (<2 km between them), associated with the name Reumén, but here we show only the one where the presence of *E.vertebralis* was also reported), 81) **Naguilán**, 82) Chaihuín, 84) Tres Chiflones, 86) R.N. Valdivia, 87) Lagunas Gemelas; (Fig. 3C): 88) Lago Queñi (Argentina), 89) Lago Lácar (Argentina), 90) Baños de Queñi (Argentina), 92) **Cerro Mirador (Cordillera Pelada)**, 93) Camino a P.N. Alerce Costero, 94) La Barra, 96) Namun Lahual, 97) Lago Espejo (Argentina), 98) Ruca Malén (Argentina), 99) Pucatrihue, 100) Bahía Mansa, 101) P.N. Puyehue, 102) Antillanca, 103) Huellelhue, 104) Catrihuala (Puente La Herradura), 105) Rupanco, 106) La Picada, 107) Puerto Blest (Argentina), 108) Arroyo Patiruco (Argentina), 109) Punta Huano (P.N. Vicente Pérez Rosales), 110) Río Manzano (P.N. Vicente Pérez Rosales), 111) Lago Fonck (Argentina), 112) Sarao, 113) Llico Bajo, 114) Río Blanco, 115) Río Correntoso, 116) P.N. Alerce Andino, 117) Ralún, 118) Río Rollizo, 119) Lago Martín (Argentina), 120) El Manso (Argentina), 121) Lenca, 122) Guabún (Punta Huechucucui), 123) Caulín, 124) Coquiao, 125) Chepu, 126) Puntra, 127) Lago Puelo (Argentina), 128) Los Hitos (Argentina), 129) Arroyo Melo (Argentina), 130) Metahue (Isla Butachauques), 131) Quetalco, 132) San Juan, 133) Mocopulli, 134) Abtao, 135) Castro, 136) Isla Alao, 137) Arroyo Torrecillas (Argentina), 138) near the mouth of the creek Zanjón Hondo (Argentina), 139) Lago Futalaufquén (Argentina), 140) Cucao, 141) Huillinco, 142) Terao, 143) Caleta Tenedor (Isla Talcán), 144) Pumalín, 145) El Amarillo, 146) Lago Amutui Quimei (Argentina), 147) Yaldad, 148) Futaleufú, 149) Río Chico, 150) Villa Santa Lucía, 151) Palena, 152) Isla Guafo, 153) Raúl Marín Balmaceda, 154) La Junta, 155) Lago Verde, 156) Puyuhuapi, 157) Queulat, 158) Lago Yulton, 159) Puerto Aguirre, 160) Isla Vergara, 161) Isla Chaculay, 162) Puerto Aysén, 163) Isla Rivero, 164) Fiordo Quitralco, 165) Isla Guerrero, 166) Puente Traihuanca, 167) Bahía Murta, 168) Área del Glaciar, 169) Canal de Ofqui, 170) Área de San Quintín, 171) Puerto Almirante Barroso, 172) Puerto Bertrand, 173) Tortel, 174) Laguna Caiquenes, 175) Isla Berta, 176) Isla Merino Jarpa, 177) Isla San Juan Stuven, 178) Lago Quetru, 179) Seno Huemules, 180) Bahía James, 181) Seno Edimburgo, 182) Puerto Edén, 183) Puerto Río Frío, 184) Bahía Broome.

*Eupsophusvertebralis* (Fig. 3D): 17) Ramadillas, 22) Los Lleulles, 24) M.N. Contulmo, 27) Tolhuaca, 57) Queule, 58) Mehuín, 63) Chanchán, 64) Llenehue, 66) Iñipulli, 67) Bosque San Martín, 185) Lingüento, 186) Pelchuquín, 187) Máfil, 70) Parque Oncol, 72) Valdivia, 73) Cuesta de Soto, 74) Huachocopihue, 75) Llancahue, 76) Los Molinos, 77) Corral, 78) Reumén, 79) Camino Viejo a La Unión, 84) Tres Chiflones, 92) Cerro Mirador (Cordillera Pelada), 94) La Barra, 96) Namun Lahual, 99) **Pucatrihue**, 100) Bahía Mansa, 103) Huellelhue, 189) Alerce 1, 104) Catrihuala (Puente La Herradura), 107) Puerto Blest (Argentina).

*Eupsophusemiliopugini* (Fig. 3D): 188) Raulintal, 99) **Pucatrihue**, 190) Piedras Negras, 191) Cerro Püschel, 106) La Picada, 192) Casa Pangue, 193) Frutillar, 109) Punta Huano (P.N. Vicente Pérez Rosales), 194) El Traiguén, 195) Lahuen Ñadi, 118) Río Rollizo, 121) Lenca, 196) Puelo, 197) Camino a Maullín, 122) Guabún, 198) Lechagua, 199) Ancud, 125) Chepu, 126) Puntra, 129) Arroyo Melo (Lago Puelo, Argentina), 140) Cucao, 200) Cucao SE, 141) Huillinco, 201) Tepuhueico, 202) Quellón, 147) Yaldad, 156) Puyuhuapi, 203) Puerto Cisnes, 204) Isla Kent, 205) Isla Melchor, 206) Caleta Vidal, 163) Puerto Yates (Isla Rivero).
